# Workforce participation in relation to cancer diagnosis, type and stage: Australian population-based study of 163,556 middle-aged people

**DOI:** 10.1007/s11764-021-01041-7

**Published:** 2021-05-18

**Authors:** Joanne Thandrayen, Grace Joshy, John Stubbs, Louise Bailey, Phyllis Butow, Bogda Koczwara, Rebekah Laidsaar-Powell, Nicole M. Rankin, Katie Beckwith, Kay Soga, Amelia Yazidjoglou, Muhammad Shahdaat Bin Sayeed, Karen Canfell, Emily Banks

**Affiliations:** 1 National Centre for Epidemiology and Population Health, Research School of Population Health, Australian National University, Canberra, Australian Capital Territory Australia; 2 CanSpeak, Sydney, New South Wales, Australia; 3 Primary Care Collaborative Cancer Clinical Trials Group Community Advisory Group, Melbourne, Victoria, Australia; 4 Psycho-oncology Cooperative Research Group Community Advisory Group, Camperdown, New South Wales, Australia; 5 The University of Sydney, Sydney, New South Wales Australia; 6 Flinders University and Flinders Medical Centre, Adelaide, South Australia, Australia; 7 The Daffodil Centre, The University of Sydney, Sydney, New South Wales Australia; 8 Prince of Wales Clinical School, University of New South Wales, Sydney, New South Wales Australia; 9 Sax Institute, Glebe, New South Wales Australia

**Keywords:** Cancer diagnosis, Cancer type, Clinical characteristics, Workforce participation, Cohort studies

## Abstract

**Purpose:**

To quantify the relationship of cancer diagnosis to workforce participation in Australia, according to cancer type, clinical features and personal characteristics.

**Methods:**

Questionnaire data (2006–2009) from participants aged 45–64 years (*n*=163,556) from the population-based 45 and Up Study (*n*=267,153) in New South Wales, Australia, were linked to cancer registrations to ascertain cancer diagnoses up to enrolment. Modified Poisson regression estimated age- and sex-adjusted prevalence ratios (PRs) for non-participation in the paid workforce—in participants with cancer (*n*=8,333) versus without (*n*=155,223), for 13 cancer types.

**Results:**

Overall, 42% of cancer survivors and 29% of people without cancer were out of the workforce (PR=1.18; 95%CI=1.15–1.21). Workforce non-participation varied substantively by cancer type, being greatest for multiple myeloma (1.83; 1.53–2.18), oesophageal (1.70; 1.13–2.58) and lung cancer (1.68; 1.45–1.93) and moderate for colorectal (1.23; 1.15–1.33), breast (1.11; 1.06–1.16) and prostate cancer (1.06; 0.99–1.13). Long-term survivors, 5 or more years post-diagnosis, had 12% (7–16%) greater non-participation than people without cancer, and non-participation was greater with recent diagnosis, treatment or advanced stage. Physical disability contributed substantively to reduced workforce participation, regardless of cancer diagnosis.

**Conclusions:**

Cancer survivors aged 45–64 continue to participate in the workforce. However, participation is lower than in people without cancer, varying by cancer type, and is reduced particularly around the time of diagnosis and treatment and with advanced disease.

**Implications for Cancer Survivors:**

While many cancer survivors continue with paid work, participation is reduced. Workforce retention support should be tailored to survivor preferences, cancer type and cancer journey stage*.*

**Supplementary Information:**

The online version contains supplementary material available at 10.1007/s11764-021-01041-7.

## Introduction

The number of cancer survivors is increasing worldwide, largely due to improvements in detection and treatment, and an aging population. In 2018, there were an estimated 43.8 million cancer survivors diagnosed within the previous 5 years globally and an estimated 0.8 million people in Australia are living with cancer [1].

People living with cancer have identified social and economic participation, and other ‘person-centred’ outcomes—such as maintaining mental health, physical ability and quality of life—as central to their ability to lead rich, fulfilling and successful lives [2, 3]. Workforce participation is a key contributor to economic and social engagement and is viewed by cancer survivors as having intrinsic and extrinsic value, providing an internal sense of self-worth and meaning, as well as having external worth through economic benefit and contribution to society [4]. Some survivors fear discrimination and being denied work due to their diagnosis, especially those in more vulnerable positions with fewer resources; those affected report significant distress at being excluded from work [4].

While substantial numbers of cancer survivors globally are of working age (i.e. 15 to 64)—an estimated 21.4 million [1]—there is limited large-scale evidence about the relationship of cancer to workforce participation. The evidence that is available indicates that work discontinuation or interruption is increased in cancer survivors compared to people without cancer; there is also suggestive evidence that it is related to the type of cancer, time since cancer diagnosis and stage of the disease, with participation being lower in those diagnosed recently and those with advanced disease [5–11]. The relationship between cancer treatment and workforce participation, and the potential role of physical disability in workforce participation among cancer survivors are underexplored in international studies. The few Australian studies that have studied work cessation [12, 13] in cancer survivors involved single cancer types and small samples. Hence, detailed information about the relationship of workforce engagement to specific cancer types, cancer stage, changes over time and treatment is lacking in Australia. Comparisons with the general population without cancer—and potentially with other health conditions—are also generally not available. This means that work-related expectations for cancer survivors and those caring for them are unclear and critical life decisions are being hampered by limited evidence. It also means the capacity of cancer survivors to continue in the workforce, if they wish to do so, may be underestimated and evidence to challenge the stigma contributing to this is inadequate.

This study aimed to comprehensively quantify workforce participation in people with cancer, overall and according to cancer type, time since diagnosis, stage and recent treatment for cancer, and to compare it with participation in people without cancer. We also aimed to examine whether the relationship between cancer and workforce participation varied by socio-demographic and health factors, and in particular, by physical functioning limitations—as a measure of co-existing physical disability. Although both paid and unpaid work were considered, paid work was emphasised due to its economic consequences. Hours of paid work per week among those in paid workforce and retirement due to ill health among those who retired were compared for people with cancer and those without cancer.

## Materials and methods

The Sax Institute’s 45 and Up Study is a population-based cohort study of 267,153 men and women aged 45 and over in the state of New South Wales (NSW), Australia. Residents in NSW were randomly sampled from the Medicare Australia database which covers all citizens and permanent residents, in addition to some temporary residents and refugees who receive universal health care coverage under Medicare. The cohort includes approximately 10% of NSW residents in the eligible age group. Individuals joined the study by completing a self-administered postal questionnaire (distributed from 1 January 2006 to 31 December 2008) and giving informed consent for long-term follow-up and linkage of their data to other population health databases. The general study methods are described in detail elsewhere [14]. Ethical approval for the conduct of the 45 and Up Study was provided by the University of New South Wales Human Research Ethics Committee. Ethical approval for the present analysis was provided by the NSW Population & Health Services Research Ethics Committee (12/CIPHS/31) and the Australian National University Human Research Ethics Committee (2012/504).

Baseline questionnaire data included self-reported information on socio-demographic factors, work and retirement, medical and surgical history, and health factors. The study questionnaire is available at https://www.saxinstitute.org.au/our-work/45-up-study/questionnaires/.

Questionnaire data from study participants were linked probabilistically to administrative datasets including data from the NSW Central Cancer Registry (CCR, 1 Jan 1994–31 Dec 2013). This probabilistic matching is conducted by the NSW Centre for Health Record Linkage (CHeReL) and is known to be highly accurate (false-positive and false-negative rates <0.4%) [15]. The linked CCR data comprised records of all diagnosed cancers (except those C44 codes that indicate a basal cell carcinoma or a squamous cell carcinoma, as these cancers are not notifiable diseases and hence not reported to cancer registries) for NSW residents, including date of diagnosis and International Classification of Diseases (ICD) coded cancer types and sites. Following the exclusion of participants with invalid data on age or date of recruitment (*n*=461, 0.17%) or data linkage errors (*n*=187, 0.07%), the analysis dataset initially consisted of 266,505 participants. Participants who were 65 and above were also excluded (*n*=102,949, 38.54%). The final analysis dataset consisted of 163,556 participants.

### Exposure

The main exposure was cancer diagnosis up to completion of the baseline questionnaire. Participants were classified as having a cancer diagnosis if they had a record in the CCR database in the 12 years prior to baseline; these individuals were considered ‘cancer survivors’. This maximised the available data from 1994 onwards and allowed the same look-back period to give equal probability of cancer identification for all participants. For those participants identified with a cancer diagnosis, the type, date of diagnosis and stage of cancer were also ascertained from the CCR database. To ensure sufficient sample sizes for reliable evidence covering as many cancer survivors as possible, we conducted specific analyses on the 12 cancer types with highest age-standardised incidence in Australia [16] a priori, except cancer of the pancreas which was excluded due to a small number of cases in the 45 and Up Study; oesophageal cancer and multiple myeloma were also included due to their known adverse effects on wellbeing [17]. Cancer types were classified as breast (ICD-10AM diagnosis code C50, women only); prostate (C61, men only); lung (C33–C34); melanoma (C43); colorectal (C18–C20); non-Hodgkin’s lymphoma (NHL, C82–C86); kidney (C64); oesophagus (C15); uterus (C54–C55, women only); bladder (C67); thyroid (C73); leukaemia (C91–C95); multiple myeloma (C90.0); and other cancers (Supplementary file, Table S1).

Time since diagnosis was classified as <1 year, 1–<5 years, 5–<10 years and 10 or more years. For participants with more than one record of cancer diagnosis, the diagnosis closest to study enrolment date was used. Stage of cancer was classified as localised to tissue or origin, regional spread to adjacent organs and/or regional lymph nodes, distant metastases, and unknown stage (only solid cancers (ICD-10AM diagnosis codes C00.0-C43.9 or C45.0-C80) were staged). Recent treatment was classified as yes/no based on responses to the baseline survey question ‘In the last month have you been treated for cancer?’ Patients were not asked whether treatment was curative or palliative, so this variable provides an indication of general treatment for cancer. The reference group of the study comprised respondents with no record of a cancer diagnosis in the CCR database prior to enrolment in the study.

### Outcomes

In our study, we examined three main outcomes: participation or non-participation in the paid workforce, number of paid hours of work per week among those in paid workforce, and retirement due to ill health or for other reasons among those retired. All outcomes were based on questions from the baseline questionnaire, ‘What is your current work status?’; ‘If you are partially or completely retired, why did you retire?’; and ‘About how many hours each week do you usually spend doing the following? paid work, voluntary/unpaid work’.

Participants classified as being in the paid workforce included those who reported valid non-zero paid hours (above 0 and below 100 hours per week) and/or current work status in at least one of the following four response options, ‘in full time paid work’, ‘in part time paid work’, ‘partially retired’ and ‘self-employed’. Participants who identified their current work status as ‘completely retired/pensioner’ or ‘disabled/sick’ or ‘doing unpaid work’ or ‘studying’ or ‘looking after home/family’ or ‘unemployed’ or ‘other’ were classified as not being in the paid workforce. Among those who retired, reasons for retirement were categorised into retirement due to ill health and retirement due to other reasons. The latter included the following response options, ‘reached usual retirement age’, ‘lifestyle reasons’, ‘to care for family member/friend’, ‘made redundant’, ‘could not find a job’ and ‘other’.

### Other variables

Socio-demographic characteristics of interest derived from the baseline questionnaire included age (categorised as 45–49 years; 50–54 years; 55–59 years; 60–64 years), sex, education (no school certificate, certificate/diploma/trade, university degree) and country of birth (Australian born, not Australian born). Region of residence, derived from the postcode’s mean Accessibility Remoteness Index of Australia Plus score [18], was categorised as major city, inner regional, outer regional and remote/very remote. Health characteristics of interest included body mass index (BMI, in kg/m^2^: underweight (15–<18.5), normal (18.5–<25), overweight (25–<30), obese (≥30–50)), physical activity (tertiles of sessions per week weighted for intensity), smoking status (never/past/current smoker), number of alcoholic drinks per week (0, 1–14, ≥15 drinks per week), physical functioning limitations measured using the Medical Outcomes Study Physical Functioning (MOS-PF) score [19] (no limitations (MOS-PF score=100), minor limitations (90–99), moderate limitations (60–89), severe limitations (<60)), psychological distress measured using the Kessler-10 (K10) scale [20] (low distress (10–<16), moderate distress (16–<22), high distress (22–50) , self-rated health (excellent/very good/good/fair/poor), and self-rated quality of life (excellent/very good/good/fair/poor). Comorbidities (yes/no) were based on responses to questions on ‘Has a doctor ever told you that you have…’

### Statistical methods

Descriptive statistics were used to summarise demographic and clinical data. Modified Poisson regression modelling [21], which combines a log Poisson regression model with robust variance estimation, was used to estimate the prevalence ratios (PRs) and their related 95% confidence intervals (CIs) to quantify the association between a cancer diagnosis and binary outcomes: non-participation in paid workforce and retirement due to ill health, overall and according to cancer types. Generalised linear models assuming a Normal distribution was used to estimate mean of paid work hours per week. Models were adjusted for age and sex (where applicable); those with no cancer were used as the reference group in both models. Further statistical adjustments were not done as the objective was to quantify and compare workforce participation among those with and without cancer in this population-based sample, rather than to establish causality between cancer and workforce participation. Prevalence ratios for non-participation in the paid workforce were also estimated stratified by clinical characteristics (time since diagnosis, stage and recent treatment), sex and age. Analyses were also conducted to examine the relationship of a prior diagnosis of cancer to workforce participation, considering physical functioning. Prevalence of workforce participation in various socio-demographic population subgroups was also measured and compared using interaction tests. Those with missing data on paid workforce (*n*=402; 0.25%) and invalid paid hours (*n*=47; 0.03%) were excluded from the corresponding analyses.

Sensitivity analyses included adjusting age on a continuous scale for the quantification of non-participation in the paid workforce, repeating analyses on paid hours of work per week including those participants who reported zero hours of paid work per week, and additional adjustment for education and region of residence separately (as a proxy for socioeconomic status) when examining the joint relationship of cancer, physical functional limitations and workforce participation.

## Results

Study participants comprised 8,333 cancer survivors and 155,223 people without cancer of working age (45–64 years). Compared to participants without cancer, cancer survivors were generally older and had poorer physical functioning limitations, less favourable overall health and lower quality of life. Cancer survivors and people without cancer were similar with respect to other characteristics examined, including sex, levels of education, region of residence, country of birth, body mass index, level of physical activity, smoking status, alcohol intake, psychological distress and comorbidities (Supplementary file, Table S2).

Across cancer types, 63% of survivors included in the analysis had their cancers diagnosed in the 5 years prior to baseline. Patients with oesophageal and lung cancer were more likely to have been diagnosed within the previous year compared to patients with other cancers. Across cancer types, 4% of survivors had metastatic disease. Patients with colorectal cancer were more likely to have the disease with regional spread, whereas for other cancer types, most survivors had a cancer which was localised to the tissue. Across cancer types, 78% of survivors had not received cancer treatment in the past month, except for those with multiple myeloma (Supplementary file, Table S3). The proportion currently receiving treatment was 16%, 33% and 53% in people with localised disease, regional spread and distant metastases, respectively (Supplementary file, Table S4).

### Workforce participation and cancer diagnosis

Overall, 58% of people with cancer were participating in the paid workforce, with 42% in full-time employment or self-employed, compared to 71% participation among people without cancer, with 55% in full-time work or self-employed (Table 1). The age- and sex-adjusted proportion of people with cancer participating the paid workforce was 62%, compared with 68% of people without cancer (Supplementary file, Table S5). The percentage of people in full-time work declined with age and was lower in women than in men; within each 5-year age-group, the percentage in full-time work was lower for those with cancer, compared to people without cancer (Supplementary file, Figure S1).

**Table 1:** Work status and retirement patterns in the study population

	Cancer survivors (*n*=8333)	Participants without cancer (*n*=155223)	Total (*n*=163556)
In paid workforce	58% (4834)	71% (109271)	114105
In full time paid work	28% (2365)	38% (58844)	61209
Self employed	14% (1179)	17% (26333)	27512
In part time paid work	18% (1527)	19% (29855)	31382
Partially retired	6% (530)	5% (8383)	8913
Not in paid workforce	42% (3481)	29% (45568)	49049
Doing unpaid work	6% (459)	5% (8302)	8761
Completely retired/pensioner	21% (1786)	13% (20002)	21788
Studying	2% (125)	2% (3238)	3363
Looking after home/family	11% (879)	11% (17162)	18041
Disabled/sick	12% (957)	5% (8271)	9228
Unemployed	3% (249)	3% (4967)	5216
Other	2% (139)	2% (2643)	2782
Paid hours per week (for those in paid workforce)			
(0,10]	484	7907	8391
(10,20]	636	12401	13037
(20,30]	744	15500	16244
(30,40]	1773	41332	43105
(40,50]	798	21334	22132
(50,60]	289	7869	8158
(60,70]	64	1691	1755
(70,80]	31	858	889
(80,90]	9	259	268
(90,100)	4	75	79
*n*	4832	109226	114058
Mean (sd)	33.85 (15.29)	35.92 (14.89)	
Median (IQR)	37.5 (24, 40)	38.0 (25, 45)	
Retired	32% (2639)	20% (30941)	33580
Causes of retirement			
Ill health	45% (1191)	29% (8951)	10142
Reached usual retirement age	10% (260)	11% (3401)	3661
Lifestyle reasons	26% (685)	32% (9989)	10674
To care for family member/friend	11% (285)	14% (4369)	4654
Made redundant	12% (314)	12% (3696)	4010
Could not find a job	5% (124)	6% (1837)	1961
Other	10% (263)	14% (4321)	4584

Percentages were calculated excluding the 402 participants missing data on paid workforce

**Fig. 1:**
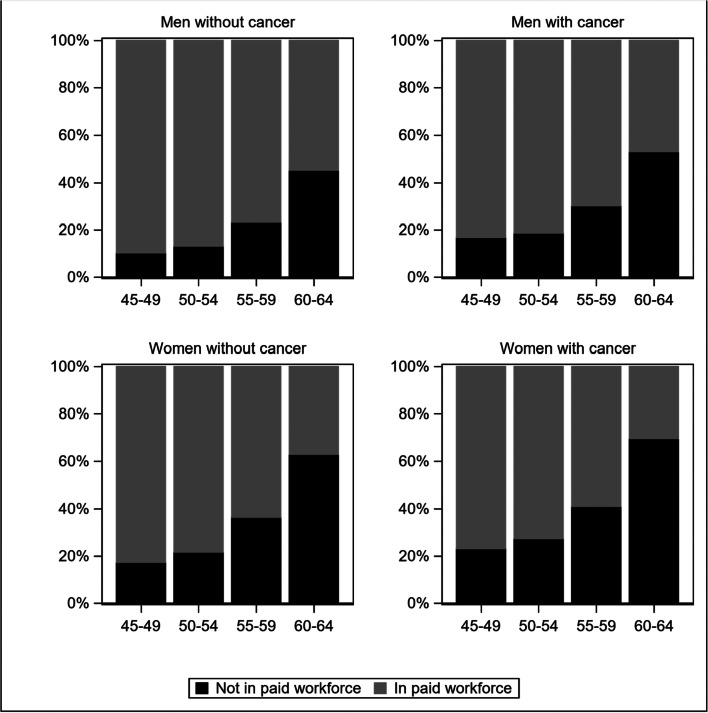
Distribution of participation in the paid workforce

**Fig. 2:**
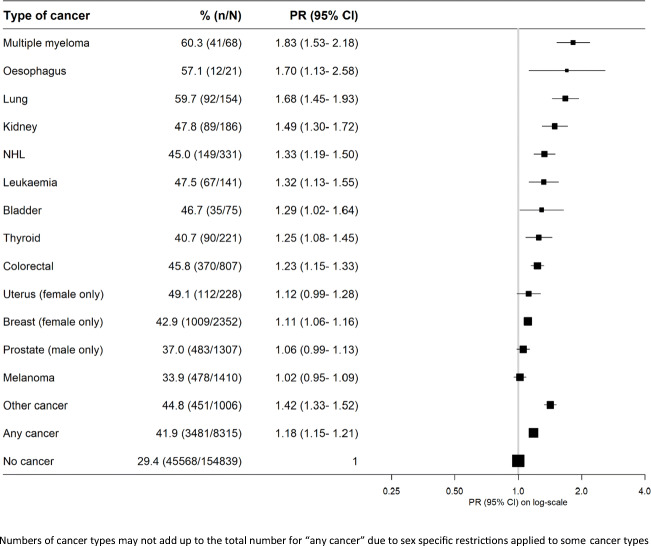
Prevalence of and adjusted (age and sex) prevalence ratios for being out of the paid workforce by cancer type

The percentage of people in the paid workforce—including full-time and part-time work—declined with age and was lower for cancer survivors than people without cancer in all age groups (Fig. 1). Cancer survivors were 18% more likely to be out of the paid workforce than cancer-free individuals (age- and sex-adjusted PR (95%CI)=1.18 (1.15–1.21)). Survivors with any cancer type, except for cancers of the uterus, prostate, and melanoma, had an elevated risk of being out of the workforce, with lung cancer, oesophageal cancer and multiple myeloma survivors having the lowest participation (Fig. 2). In general, similar patterns were noted when stratifying by sex and age (Supplementary file, Figure S2; Figure S3).

**Fig. 3:**
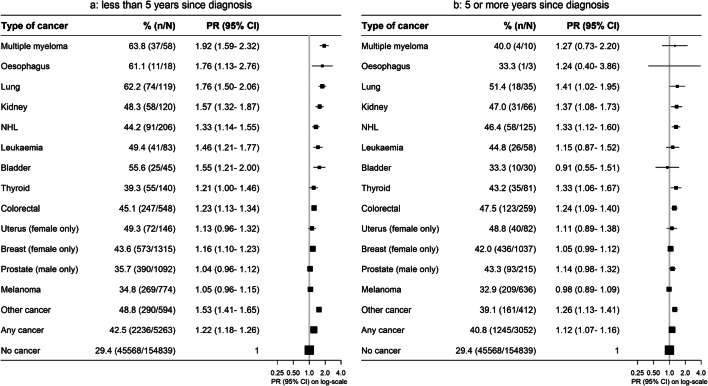
Prevalence of and adjusted (age and sex) prevalence ratios for being out of the paid workforce by cancer type and time since diagnosis. **a** Less than 5 years since diagnosis. **b** 5 or more years since diagnosis

**Fig. 4:**
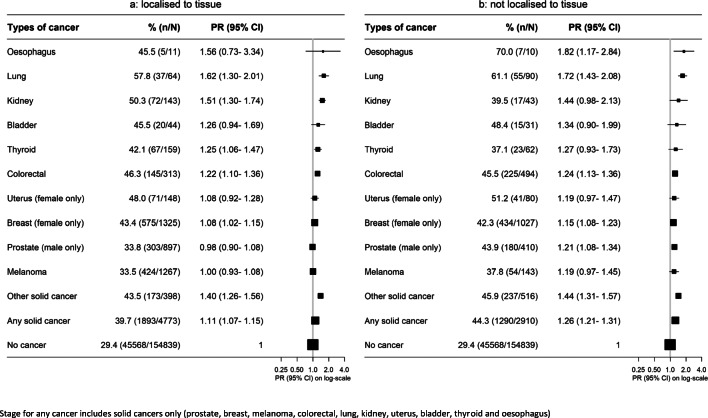
Prevalence of and adjusted (age and sex) prevalence ratios for being out of the paid workforce by cancer type and stage. **a** Localised to tissue. **b** Not localised to tissue

**Fig. 5:**
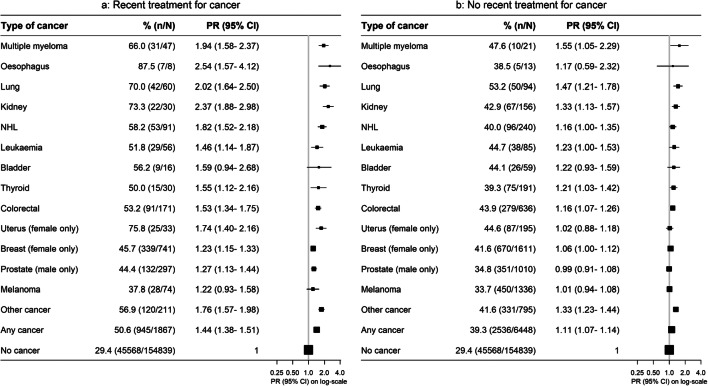
Prevalence of and adjusted (age and sex) prevalence ratios for being out of the paid workforce by cancer type and recent treatment. **a** Recent treatment for cancer. **b** No recent treatment for cancer

While cancer survivors were more likely to be out of the workforce than people without cancer, this relationship was stronger in survivors diagnosed within the last 5 years than in those diagnosed 5 or more years ago, across cancer types (Fig. 3). Workforce participation also varied by solid cancer stage, with those with localised disease having an overall 11% higher risk of being out of the workforce compared to 26% higher risk for those with non-localised disease (Fig. 4). People who reported receiving treatment within the past month had a greater elevation in the risk of being out of the workforce, compared to cancer survivors not receiving recent treatment (Fig. 5).

### Paid hours of work per week and cancer diagnosis

**Fig. 6:**
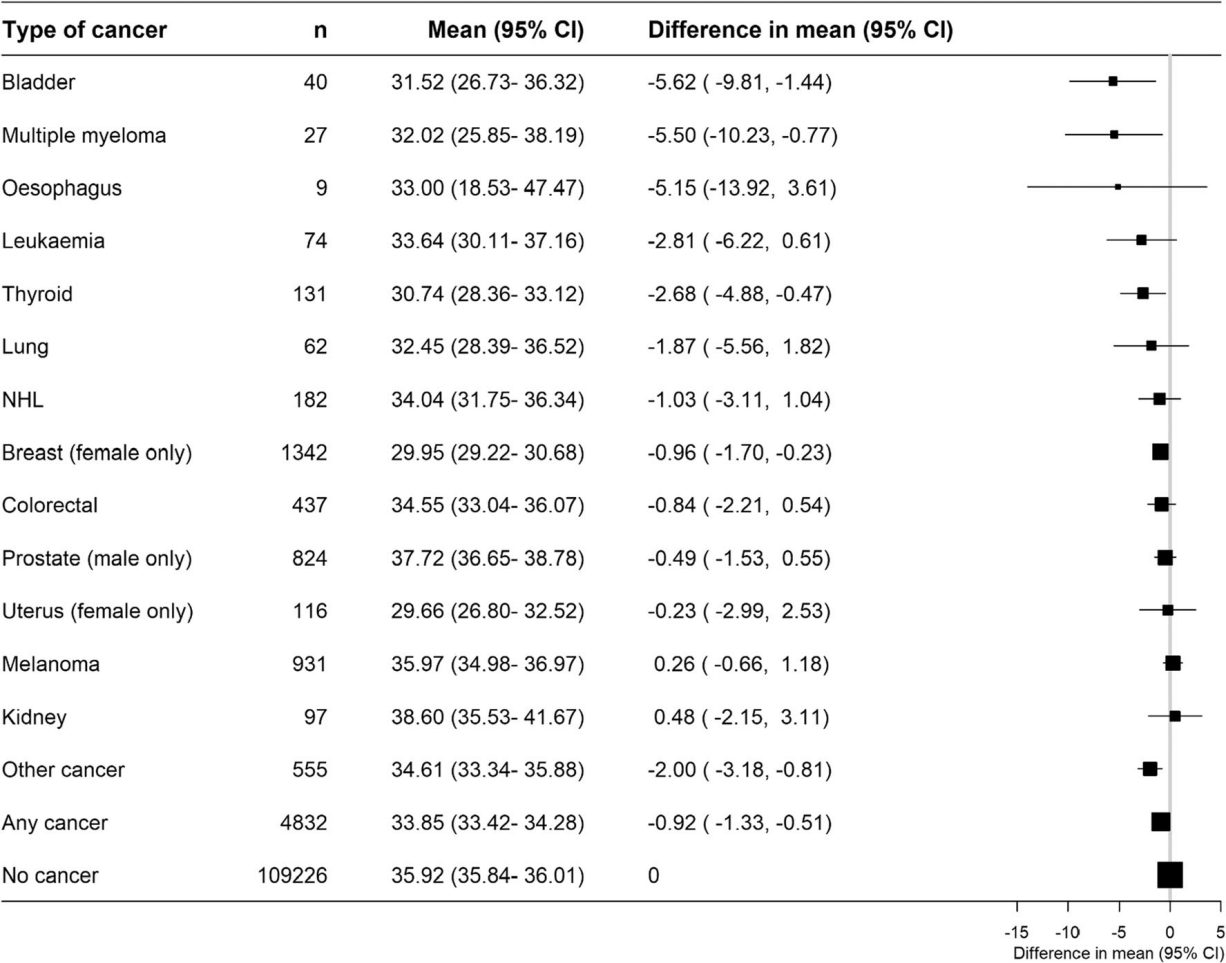
Paid hours (0<paid hours<100) of work per week (adjusted for age and sex) for cancer survivors by cancer type, in comparison to people without cancer, among those in the paid workforce

Among those in the paid workforce, average working hours per week were 33.85 (15.29) in cancer survivors and 35.92 (14.89) in those without cancer (Table 1), amounting to an average of 0.92 hours less per week (95%CI 0.51–1.33) in those with versus without cancer. Across cancer type, survivors did not, in general, have significant differences in their working hours per week, except for survivors of bladder, multiple myeloma, thyroid and breast who had lower working hours per week than people without cancer (Fig. 6).

Among people with and without cancer who were in paid work, men worked longer hours (greater than 35 hours on average) than women; cancer survivors worked fewer hours compared to people without cancer (Supplementary file, Figure S4).

### Retirement due to ill health and cancer diagnosis

Overall, 32% of people with cancer had retired, compared to 20% of people without cancer (Table 1); the percentage of people who were not working because of illness or disability was also greater in those with cancer compared to those without cancer (Supplementary file, Figure S5).

**Fig. 7:**
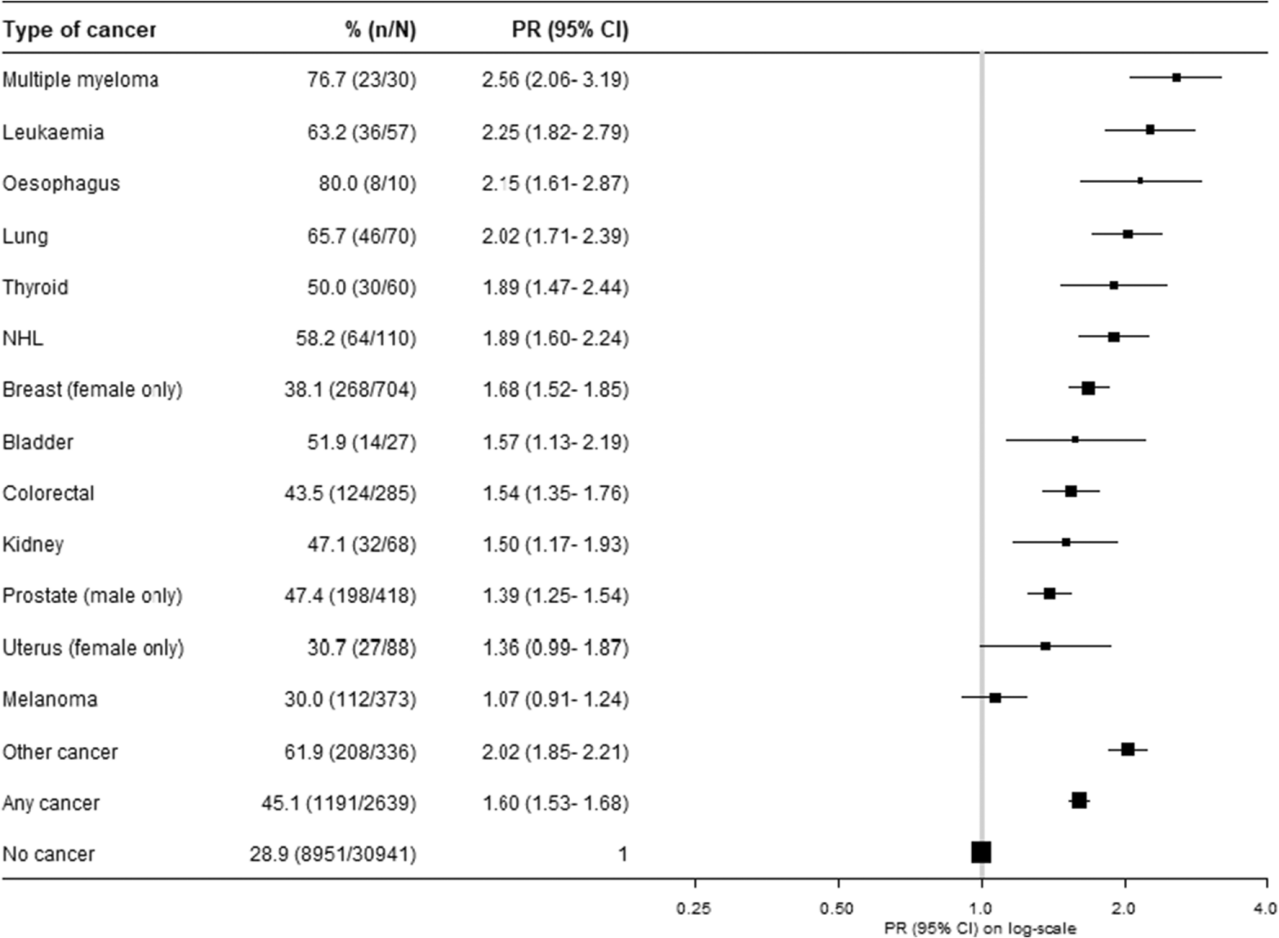
Prevalence of and adjusted (age and sex) prevalence ratios for retiring due to ill health versus retiring for other reasons by cancer type, among retired participants

Retirees with cancer were more likely to attribute their retirement to ill health than retirees without cancer (Table 1); this was seen for both men and women and across all age groups (Supplementary file, Figure S6). There was a 60% higher prevalence of retirement due to ill health (PR(95%CI)=1.60(1.53–1.68)) in people with versus without cancer overall. Across all cancer types except for cancers of the uterus and melanoma, there was a statistically significant higher prevalence of retirement due to ill health in people with versus without cancer (Fig. 7).

### Socio-demographic factors associated with workforce participation

Regardless of age, sex, region of residence, country of birth or education level, people with cancer were more likely to be out of the paid workforce in comparison to people without cancer (Supplementary file, Figure S7). The relative risk of cancer to non-participation in the paid workforce was significantly higher in younger compared to older survivors (*p*_interaction_=0.0004), men compared to women (*p*_interaction_=0.0002), and those with a university degree compared to those without school certificate (*p*_interaction_<0.0001). No significant difference was observed according to whether participants were born in Australia or elsewhere or whether they lived in the city or country. The observed higher relative risks were in terms of prevalence ratios; the baseline percentage in paid work and the absolute differences between those with and without cancer should be considered. For example, 54% of those with no qualifications were not in paid work if they did not have cancer, with 64% out of work if they had cancer. Corresponding figures for people with university degrees were 18% and 28%. Fifty-five percent of older participants (60–64) were not in paid work if they did not have cancer, with 61% out of work if they had cancer. Corresponding figures for younger participants (45–49) were 15% and 22%. Thirty-four percent of females were not in paid work if they did not have cancer, with 45% out of work if they had cancer. Corresponding figures for males were 24% and 38%.

### Workforce participation and physical limitations

**Fig. 8:**
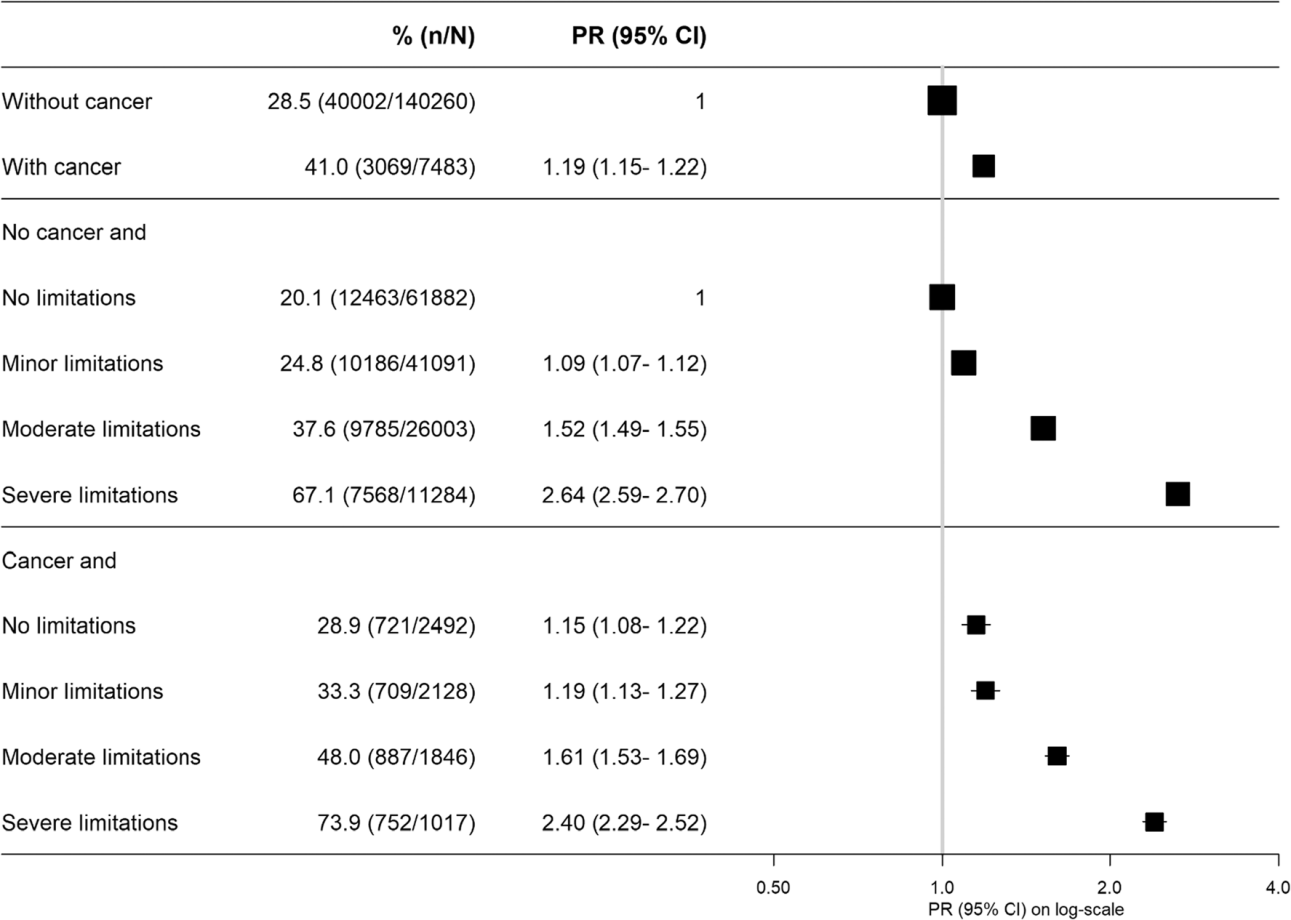
Prevalence of and adjusted (age and sex) prevalence ratios for being out of the paid workforce according to joint categories of physical functioning limitations and cancer

People with physical disability were more likely to be out of the paid workforce, regardless of whether or not they had cancer (Fig. 8). Among participants without limitations to physical functioning, participants with cancer were more likely to be out of the paid workforce than participants without cancer ((PR(95%CI)=1.15(1.08–1.22)). While the excess relative risk of being out of the paid workforce for people with versus without cancer was around 20% overall—and 15% in able-bodied individuals—people with severe limitations to physical functioning were around 140 to 160% more likely to be out of the paid workforce compared to people without physical limitations. This indicates that the relationship of physical functioning to lack of workforce participation was 7–9 times stronger than that of cancer to the same outcome.

### Sensitivity analyses

Among participants in and out of the workforce, people with cancer worked on average 2.68 hours less per week (95%CI 2.27–3.09), in comparison to those without cancer. All cancer types except for cancers of the uterus, prostate and melanoma had lower working hours per week compared to people without cancer (Supplementary file, Figure S8).

The overall pattern in quantification of non-participation in the paid workforce in cancer survivors compared to cancer-free individuals was similar when age was adjusted on a continuous scale (Supplementary file, Figure S9). Additional adjustment for education or region of residence did not materially change the findings on physical functional limitations, cancer and workforce participation (Supplementary file, Figure S10; Figure S11).

## Discussion

In this large population-based study, cancer survivors demonstrated considerable participation in the paid workforce; once age and sex were taken into account, 62% of cancer survivors and 68% of people without cancer aged 45–64 in this study reported being in paid work, and worked on average just 1 hour less than people without cancer. Nonetheless, compared to people without cancer, cancer survivors were on average around 20% less likely to participate in the paid workforce and were 60% more likely to retire due to ill health.

There was substantial variation in workforce participation according to cancer type, with the lowest participation for survivors of multiple myeloma, lung cancer and the composite group of less common “other cancers”. Participation was lowest in those with recent diagnosis and treatment, and advanced cancer stage, with longer-term survivors having an average 12% reduction in workforce participation (versus 20% for all survivors) compared to people without cancer. Cancer type appeared to have less influence on participation among longer-term survivors.

Overall, when compared to those people without cancer, cancer survivors had lower workforce participation in all of the population subgroups examined. In absolute terms, a higher percentage of less-educated cancer survivors were out of the paid workforce, as were older and female survivors. Stratification based on cancer status and physical functioning limitations showed that non-participation in the paid workforce was more strongly related to physical functioning limitations than cancer diagnosis itself, although both appeared to play a role.

The poorest outcomes (lower levels of workforce participation and higher levels of retirement due to ill health) for some cancers are likely to be linked to the severity of the cancer and their associated treatment and physical side effects. For instance, survivors of multiple myeloma and a number of rare cancers such as sarcomas included in the “other” cancer group tend to undergo more intense and toxic treatment. Survivors of haematological cancers (including multiple myeloma) have relatively longer survival but their participation in the workforce seems to be disproportionately impacted, potentially due to factors such as fatigue [22]. Lung cancer is typically diagnosed at an advanced stage when people experience symptoms that are debilitating, either as a result of the disease itself, co-morbidity such as chronic obstructive pulmonary disease or side-effects of cancer treatments [23, 24]. People with lung cancer also experience a particularly high symptom burden—fatigue, pain, breathlessness—which may make paid employment more difficult [25]. There is substantial heterogeneity of employment experience according to cancer type but lack of evidence as to the relative contributions of disease burden/stage, type of treatment or comorbidities; our data indicate that all are likely to play a role. Furthermore, little is known about the quality of employment faced by cancer survivors and this is likely to be a fruitful area of future research.

In general, paid working hours per week among people with most cancer types were similar to the number of hours for people without cancer, except for multiple myeloma and cancers of the bladder and thyroid where survivors worked fewer hours per week. This may be explained by cancer-specific physical side effects such as passing urine frequently, difficulty concentrating or fatigue in survivors of cancers of the bladder [26] and thyroid [27].

Our study provides the most comprehensive analysis of cancer and workforce to date and is also the only one, to our knowledge, to examine the potential role of physical disability in workforce participation among cancer survivors, finding that cancer survivors with few limitations to physical functioning have participation similar to people without cancer. Future studies are planned to examine the role of other symptoms, such as pain, in cancer survivorship. Furthermore, very few international studies have focused on retirement due to ill health and working hours per week. To our knowledge, the only study that explored aspects of retirement due to ill health was in Denmark [6]. That study showed an increased likelihood of disability pension after a cancer diagnosis. A study in the USA [10] showed that female cancer survivors worked 3.5 hours less per week (est= −3.536, *p*<0.05) whereas male cancer survivors worked 5.7 less hours per week (est= −5.657, *p*<0.05) than people without cancer.

Given the highly variable nature of work from country to country, as well as differing cancer survival and management, it is important to have large-scale evidence to inform local and global considerations. To our knowledge, this is the first Australian study to examine and compare the levels of workforce participation and retirement due to ill health among those with and without cancer at scale. The ascertainment of cancer diagnoses from linked cancer registry data was comprehensive and identified a sufficient number of cancer type and some of the rarer cancers for inclusion in the study. The large size of the population of the study has also allowed for stratification by clinical characteristics and population subgroups.

Our findings add to those of previous international studies which demonstrated that having cancer is associated with lower levels of workforce participation, with general reduced participation with recent cancer diagnosis and advanced cancer stage. A study of cancer survivors from South Korea [8] found that, adjusting for age, sex and education, survivors were more than 2 times more likely to not be working (aOR=2.29, 95%CI 2.26–2.32) and less likely to be in paid work (aOR=0.74 95%CI 0.72–0.75) compared to people without cancer. A Finnish study [11] found that cancer survivors were less likely to be employed than cancer-free individuals (RR=0.91, 95%CI=0.90–0.92) and the greatest risk was for lung (RR=0.63, 95%CI=0.56–0.71), multiple myeloma (RR=0.67, 95%CI=0.54–0.83), nervous system (RR=0.72, 95%CI=0.69–0.75) and head and neck cancer (RR=0.80, 95%CI=0.74–0.86) (adjustment not specified). Our study focuses on 13 cancer types and provides a quantitative evaluation of non-participation in the workforce for each cancer type, overall, and according to time since diagnosis, stage and recent treatment for cancer.

Our definition of workforce participation, including full-time or part-time work, was based on participants’ perception of their full-time or part-time work status. While this approach helps to minimise recall problems [28], we cannot rule out recall problems in the study, particularly in self-reported paid work hours per week. For instance, participants may not recall their exact number of paid work hours per week. Participants in cohort studies are generally healthier and more health conscious than the source population, as in the case of the 45 and Up Study [29]. This means that caution should be applied in generalising from the absolute estimates of prevalence in this study. However, relative estimates based on internal comparisons with the cohort—such as the prevalence ratios presented here—are likely to remain valid and generalisable [29, 30]. It is not possible to establish that the outcomes measured in the study were solely due to a cancer diagnosis; they could have been present before the cancer diagnosis itself. However, the comparison group was defined only as not having cancer and would include people with comorbidity other than cancer. It was not possible to establish the extent to which workforce participation represented an active and positive choice of the survivor or an unwelcome outcome—including being forced out of work due to concerns about capacity, or being forced to continue with work due to economic necessity. Our study did not investigate the type of work performed by participants as this information was not available in the study questionnaire. Our study was restricted to middle-aged people, the vast majority of workforce participants affected by cancer; we are not able to draw conclusions about younger cancer survivors. In our study, recent treatment was based on responses to the question on treatment received in the last month; data on treatments received prior to the last month were not available. The comprehensive nature of the analyses presented here means that multiple statistical tests were performed; this should be considered when interpreting the findings.

This study provides large-scale, detailed empirical evidence on non-participation in the paid workforce among those with and without cancer and bridges some gaps of knowledge. This study highlights that cancer does not have uniform employment impacts, with the majority of long-term survivors, particularly those without disability, having participation only slightly lower than people without cancer. It highlights specific subgroups where participation is markedly lower—focusing attention on these groups (multiple myeloma, lung, those currently undergoing treatment) may be of value. It also identifies subgroups of cancer survivors who might be more at risk of leaving the workforce for the development of interventions or for policy and practice change. This large-scale evidence may assist cancer survivors to have a better understanding of likely participation in the paid workforce during the cancer journey and thus inform their expectations and lifestyle accordingly. It may also assist to identify better ways of supporting those living with cancer throughout their journey to recovery and beyond. This study provides insights to cancer survivors and their respective employers about average working hours per week and retirement due to ill health. This may lead to better planning throughout the cancer journey.

## Conclusion

Participation in the paid workforce among cancer survivors was substantial, with the majority in this study in part-time or full-time paid work, and participation in long-term survivors without physical disability being only slightly lower than that in people without cancer. However, overall participation was lower than that in people without cancer, and cancer survivors were also more likely to retire due to ill health and have reduced working hours. Workforce participation varied by cancer type and stage in the cancer journey; it was lowest for survivors of multiple myeloma, lung cancer and for those with recent cancer diagnosis, recent cancer treatment and advanced cancer stage. Workforce participation was reduced in all population subgroups examined. Physical disability was a major contributor to non-participation in the paid workforce, suggesting the importance of rehabilitation and the need to integrate considerations of the role of chronic diseases, including cancer, on social policies. Workforce retention support should be tailored to survivor preferences and individual circumstances, taking into consideration factors such as cancer type and cancer journey stage*.*

## Supplementary information

ESM 1 (DOCX 4833 kb)

## References

[1] International Agency of Research on Cancer. Global Cancer Observatory: World Health Organization; 2018. https://gco.iarc.fr/

[2] Hodgkinson K, Butow P, Hunt GE, Pendlebury S, Hobbs KM, Wain G. Breast cancer survivors’ supportive care needs 2–10 years after diagnosis. Support Care Cancer. 2007;15(5):515–23. 10.1007/s00520-006-0170-2 17120068

[3] Jefford M, Karahalios E, Pollard A, Baravelli C, Carey M, Franklin J, et al. Survivorship issues following treatment completion—results from focus groups with Australian cancer survivors and health professionals. J Cancer Survivorship. 2008;2(1):20–32. 10.1007/s11764-008-0043-4 18648984

[4] Butow P, Laidsaar-Powell R, Konings S, Lim CYS, Koczwara B. Return to work after a cancer diagnosis: a meta-review of reviews and a meta-synthesis of recent qualitative studies. J Cancer Survivorship. 2020;14(2):114–34. 10.1007/s11764-019-00828-z 31858379

[5] Rottenberg Y, Amir Z, De Boer A. Work cessation after cancer diagnosis: a population-based study. Occup Med. 2019;69(2):126–32. 10.1093/occmed/kqz013 30882861

[6] Heinesen E, Imai S, Maruyama S. Employment, job skills and occupational mobility of cancer survivors. J Health Econ. 2018;58:151–75. 10.1016/j.jhealeco.2018.01.006 29486331

[7] Jeon SH. The long-term effects of cancer on employment and earnings. Health Econ. 2017;26(5):671–84. 10.1002/hec.3342 27045223

[8] Lee MK, Yun YH. Working situation of cancer survivors versus the general population. J Cancer Survivorship. 2015;9(2):349–60. 10.1007/s11764-014-0418-7 25492237

[9] Torp S, Nielsen RA, Fosså SD, Gudbergsson SB, Dahl AA. Change in employment status of 5-year cancer survivors. Eur J Public Health. 2013;23(1):116–22. 10.1093/eurpub/ckr192 22227027

[10] Moran JR, Short PF, Hollenbeak CS. Long-term employment effects of surviving cancer. J Health Econ. 2011;30(3):505–14. 10.1016/j.jhealeco.2011.02.001 PMC3110504 21429606

[11] Taskila-Åbrandt T, Pukkala E, Martikainen R, Karjalainen A, Hietanen P. Employment status of Finnish cancer patients in 1997. Psycho-Oncology: J Psychol Soc Behav Dimens Cancer. 2005;14(3):221–6. 10.1002/pon.838 15386773

[12] Gordon LG, Beesley VL, Mihala G, Koczwara B, Lynch BM. Reduced employment and financial hardship among middle-aged individuals with colorectal cancer. Euro J Cancer Care. 2017;26(5):e12744. 10.1111/ecc.12744 28771857

[13] Beesley VL, Vallance JK, Mihala G, Lynch BM, Gordon LG (2017). Association between change in employment participation and quality of life in middle-aged colorectal cancer survivors compared with general population controls. Psycho-oncology..

[14] Banks E, Redman S, Harris M, Sitas F, Smith W, Taylor L, et al. Cohort profile: the 45 and up study. Int J Epidemiol. 2008;37(5):941–7. 10.1093/ije/dym184 PMC2557061 17881411

[15] Centre for Health Record Linkage. Quality assurance. CHeReL. 2020. https://www.cherel.org.au/quality-assurance.

[16] Australian Institute of Health and Welfare (2019). Cancer in Australia 2019.

[17] Kent EE, Ambs A, Mitchell SA, Clauser SB, Smith AW, Hays RD (2015). Health-related quality of life in older adult survivors of selected cancers: data from the SEER-MHOS linkage. Cancer..

[18] Australian Institute of Health and Welfare (2004). Rural, regional and remote health: a guide to remoteness classifications.

[19] Hays RD, Liu H, Spritzer K, Cella D. Item response theory analyses of physical functioning items in the medical outcomes study. Med Care. 2007;45:S32–S8. 10.1097/01.mlr.0000246649.43232.82 17443117

[20] Kessler RC, Andrews G, Colpe LJ, Hiripi E, Mroczek DK, Normand S-L, et al. Short screening scales to monitor population prevalences and trends in non-specific psychological distress. Psychol Med. 2002;32(6):959–76. 10.1017/s0033291702006074 12214795

[21] Zou G. A modified Poisson regression approach to prospective studies with binary data. Am J Epidemiol. 2004;159(7):702–6. 10.1093/aje/kwh090. 10.1093/aje/kwh090 15033648

[22] McGrath PD, Hartigan B, Holewa H, Skarparis M. Returning to work after treatment for haematological cancer: findings from Australia. Support Care Cancer. 2012;20(9):1957–64. 10.1007/s00520-011-1298-2 22033835

[23] Ellis J (2012). The impact of lung cancer on patients and carers. Chronic respiratory disease..

[24] Iyer S, Roughley A, Rider A, Taylor-Stokes G. The symptom burden of non-small cell lung cancer in the USA: a real-world cross-sectional study. Supportive Care in Cancer : Official Journal of the Multinational Association of Supportive Care in Cancer. 2013;22(1):181–7. 10.1007/s00520-013-1959-4. 10.1007/s00520-013-1959-4 24026981

[25] Yang P, Cheville AL, Wampfler JA, Garces YI, Jatoi A, Clark MM, et al. Quality of life and symptom burden among long-term lung cancer survivors. J Thorac Oncol. 2012;7(1):64–70. 10.1097/JTO.0b013e3182397b3e. 10.1097/JTO.0b013e3182397b3e PMC3241852 22134070

[26] Edmondson AJ, Birtwistle JC, Catto JWF, Twiddy M. The patients’ experience of a bladder cancer diagnosis: a systematic review of the qualitative evidence. J Cancer Survivorship : Res Pract. 2017;11(4):453–61. 10.1007/s11764-017-0603-6. 10.1007/s11764-017-0603-6 PMC5500680 28213769

[27] Sawka AM, Goldstein DP, Brierley JD, Tsang RW, Rotstein L, Ezzat S, et al. The impact of thyroid cancer and post-surgical radioactive iodine treatment on the lives of thyroid cancer survivors: a qualitative study. PloS One. 2009;4(1):e4191-e. 10.1371/journal.pone.0004191. 10.1371/journal.pone.0004191 PMC2615133 19142227

[28] Australian Bureau of Statistics. Employment. In: 6102.0.55.001 - Labour statistics: concepts, sources and methods, Feb 2018 2020. https://www.abs.gov.au/ausstats/abs@.nsf/Lookup/by%20Subject/6102.0.55.001~Feb%202018~Main%20Features~Employment~4.

[29] Mealing NM, Banks E, Jorm LR, Steel DG, Clements MS, Rogers KD. Investigation of relative risk estimates from studies of the same population with contrasting response rates and designs. BMC Med Res Methodol. 2010;10(1):26. 10.1186/1471-2288-10-26 PMC2868856 20356408

[30] Rothman KJ, Gallacher JE, Hatch EE. Why representativeness should be avoided. Int J Epidemiol. 2013;42(4):1012–4. 10.1093/ije/dys223 PMC3888189 24062287

