# Supplementary material for: Workforce participation in relation to cancer diagnosis, type and stage: Australian population-based study of 163,556 middle-aged people
Source: J Cancer Surviv. 2021 May 18;16(2):461–73. doi: 10.1007/s11764-021-01041-7 (PMC8964624; doi:10.1007/s11764-021-01041-7)
Supplement: Supplementary file 1 — (DOCX 4833 kb) [file 11764_2021_1041_MOESM1_ESM.docx]

**Supplementary file**

This Supplementary file provides additional information to that in the article by Joanne Thandrayen, Grace Joshy, John Stubbs, Louise Bailey, Phyllis Butow, Bogda Koczwara, Rebekah Laidsaar-Powell, Nicole M. Rankin, Katie Beckwith, Kay Soga, Amelia Yazidjoglou, Muhammad Shahdaat Bin Sayeed, Karen Canfell, and Emily Banks titled “Workforce participation in relation to cancer diagnosis, type and stage: Australian population-based study of 163,556 middle-aged people”. The article is published in the Journal of Cancer Survivorship.

**Corresponding Author:**

Dr Joanne Thandrayen

National Centre for Epidemiology and Population Health

Research School of Population Health, Australian National University

[Joanne.Thandrayen@anu.edu.au](mailto:Grace.Joshy@anu.edu.au)

**Table S1: Definition of other cancer**

| **icd-10am code** | **icd-10am label** | **n** |
| --- | --- | --- |
| C00 | Lip | 64 |
| C01-C02 | Tongue | 40 |
| C03-C06 | Mouth | 27 |
| C07-C08 | Salivary glands | 21 |
| C09-C10 | Oropharynx | 50 |
| C11 | Nasopharynx | 16 |
| C12-C13 | Hypopharynx | 5 |
| C14 | Other sites in pahrynx | 3 |
| C16 | Stomach | 50 |
| C17 | Small intestine | 33 |
| C21 | Anus | 24 |
| C22 | Liver | 22 |
| C23-C24 | Gallbladder and extrahepatic bile ducts | 13 |
| C25 | Pancreas | 22 |
| C30-C31 | Nose, sumses, etc | 9 |
| C32 | Larynx | 37 |
| C37-C39 | Other thoracic and respiratory organs | 3 |
| C40-C41 | Bone | 11 |
| C45 | Mesothelioma | 5 |
| C46 | Kaposi sarcoma | 8 |
| C48 | Peritoneum | 11 |
| C47, C49 | Other soft tissue | 39 |
| C51 | Vulva | 19 |
| C52 | Vagina | 5 |
| C53 | Cervix | 63 |
| C56 | Ovary | 96 |
| C57-C58 | Other female genital organs and placenta | 9 |
| C60 | Penis | 3 |
| C62 | Testis | 67 |
| C63 | Other male genital organs | 2 |
| C65-C66, C68 | Other urinary organs | 15 |
| C69 | Eye | 29 |
| C71 | Brain | 39 |
| C70, C72 | Other central nervous system | 5 |
| C74-C75 | Other endocrine glands | 4 |
| C76 | Other and ill-defined sites | 2 |
| C80 | Unknown primary site | 47 |
| C81 | Hodgkin's disease | 45 |
| C90.1-C90.9 | Other plasma cell | 10 |
| C96, D45, D47.1, D47.3-D47.5 | Other cancers of the blood and lymphatic system | 28 |
| D46 | Myelodyplastic syndromes | 9 |
| Total |  | 1010 |

**Table S2. Characteristics of the study population**

|  | **Cancer** | **Participants** | **Total** |
| --- | --- | --- | --- |
|  | **survivors** | **without cancer** | **(n=163556)** |
|  | **(n=8333)** | **(n=155223)** |  |
| **Age group** |  |  |  |
| 45- 49 years | 11%(892) | 22%(33970) | 34862 |
| 50-54 years | 21%(1712) | 27%(41228) | 42940 |
| 55- 59 years | 30%(2494) | 28%(42952) | 45446 |
| 60-64 years | 39%(3235) | 24%(37073) | 40308 |
| **Sex** |  |  |  |
| Male | 42%(3501) | 43%(66952) | 70453 |
| Female | 58%(4832) | 57%(88271) | 93103 |
| **Education** |  |  |  |
| No school certificate | 9%(777) | 8%(12907) | 13684 |
| Certificate/diploma/trade | 64%(5365) | 62%(96805) | 102170 |
| University degree | 25%(2100) | 28%(43958) | 46058 |
| Missing | 1%(91) | 1%(1553) | 1644 |
| **Region of residence** |  |  |  |
| Major cities | 52%(4327) | 52%(80150) | 84477 |
| Inner regional | 35%(2937) | 35%(54029) | 56966 |
| Outer regional | 10%(822) | 10%(16189) | 17011 |
| Remote/very remote | 1%(80) | 1%(1670) | 1750 |
| Missing | 2%(167) | 2%(3185) | 3352 |
| **Country of birth** |  |  |  |
| Australia | 79%(6583) | 76%(117727) | 124310 |
| Not Australia | 20%(1694) | 24%(36683) | 38377 |
| Missing | 1%(56) | 1%(813) | 869 |
| **BMI** |  |  |  |
| Underweight | 1%(97) | 1%(1580) | 1677 |
| Normal | 31%(2573) | 34%(52133) | 54706 |
| Overweight | 36%(3027) | 36%(56049) | 59076 |
| Obese | 26%(2133) | 23%(36406) | 38539 |
| Missing | 6%(503) | 6%(9055) | 9558 |
| **Physical activity** |  |  |  |
| First tertile | 31%(2618) | 29%(45330) | 47948 |
| Second tertile | 33%(2779) | 33%(51231) | 54010 |
| Third tertile | 33%(2742) | 36%(55240) | 57982 |
| Missing | 2%(194) | 2%(3422) | 3616 |
| **Smoking status** |  |  |  |
| Current smoker | 7%(619) | 10%(14793) | 15412 |
| Past smoker | 37%(3084) | 34%(52342) | 55426 |
| Never smoker | 55%(4605) | 56%(87566) | 92171 |
| Missing | 0%(25) | 0%(522) | 547 |
| **Alcohol intake (drinks per week)** |  |  |  |
| 0 | 32%(2631) | 29%(45651) | 48282 |
| 1-14 | 52%(4344) | 54%(83749) | 88093 |
| ≥15 | 15%(1241) | 15%(23538) | 24779 |
| Missing | 1%(117) | 1%(2285) | 2402 |
| **CVD** |  |  |  |
| No | 87%(7290) | 90%(140393) | 147683 |
| Yes | 13%(1043) | 10%(14830) | 15873 |
| **Diabetes** |  |  |  |
| No | 93%(7720) | 94%(145279) | 152999 |
| Yes | 7%(613) | 6%(9944) | 10557 |
| **Parkinson** |  |  |  |
| No | 100%(8293) | 100%(154823) | 163116 |
| Yes | 0%(40) | 0%(400) | 440 |
| **Asthma** |  |  |  |
| No | 77%(6398) | 75%(117014) | 123412 |
| Yes | 10%(818) | 11%(16475) | 17293 |
| Missing | 13%(1117) | 14%(21734) | 22851 |
| **Physical functioning limitations** |  |  |  |
| No limitation | 30%(2498) | 40%(62011) | 64509 |
| Minor limitation | 26%(2133) | 27%(41183) | 43316 |
| Moderate limitation | 22%(1851) | 17%(26069) | 27920 |
| Severe limitation | 12%(1018) | 7%(11329) | 12347 |
| Missing | 10%(833) | 9%(14631) | 15464 |
| **Psychological distress** |  |  |  |
| Low distress | 68%(5682) | 70%(108079) | 113761 |
| Moderate distress | 17%(1441) | 16%(25563) | 27004 |
| High distress | 8%(647) | 8%(13061) | 13708 |
| Missing | 7%(563) | 5%(8520) | 9083 |
| **Self-rated health** |  |  |  |
| Excellent | 11%(919) | 18%(28261) | 29180 |
| Very good | 33%(2764) | 38%(59423) | 62187 |
| Good | 35%(2940) | 30%(46480) | 49420 |
| Fair | 14%(1181) | 9%(14299) | 15480 |
| Poor | 3%(271) | 2%(2573) | 2844 |
| Missing | 3%(258) | 3%(4187) | 4445 |
| **Self-rated quality of life** |  |  |  |
| Excellent | 21%(1758) | 27%(41933) | 43691 |
| Very good | 36%(2987) | 37%(57883) | 60870 |
| Good | 27%(2262) | 24%(36522) | 38784 |
| Fair | 10%(834) | 7%(10523) | 11357 |
| Poor | 2%(132) | 1%(2195) | 2327 |
| Missing | 4%(360) | 4%(6167) | 6527 |

**Table S3. Clinical characteristics of cancer by cancer type**

|  | Bladder | Breast | Colorectal | Kidney | Leukaemia | Lung | Melanoma | Multiple myeloma | NHL | Oesophagus | Prostate | Thyroid | Uterus | Other cancer | Any cancer |
| --- | --- | --- | --- | --- | --- | --- | --- | --- | --- | --- | --- | --- | --- | --- | --- |
| **n** | 75 | 2364 | 810 | 186 | 141 | 155 | 1413 | 68 | 331 | 21 | 1310 | 221 | 228 | 1010 | 8333 |
| **Median time since diagnosis (years)** | 4.3 | 4.3 | 3.2 | 3.4 | 4.0 | 1.9 | 4.7 | 2.9 | 3.5 | 1.5 | 2.2 | 3.8 | 3.3 | 4.0 | 3.6 |
| **Time since diagnosis (years)** |  |  |  |  |  |  |  |  |  |  |  |  |  |  |  |
| <1 | 13.3% | 11.4% | 20.7% | 18.3% | 14.2% | 27.7% | 12.7% | 17.6% | 15.7% | 28.6% | 24.9% | 15.8% | 18.0% | 15.5% | 16.2% |
| 1 to <5 | 46.7% | 44.5% | 47.2% | 46.2% | 44.7% | 49.7% | 42.2% | 67.6% | 46.5% | 57.1% | 58.5% | 47.5% | 46.1% | 43.5% | 47.0% |
| 5 to <10 | 34.7% | 34.6% | 25.4% | 26.9% | 36.2% | 20.0% | 35.5% | 14.7% | 31.4% | 14.3% | 15.1% | 31.2% | 31.1% | 30.5% | 29.4% |
| 10 or more | 5.3% | 9.4% | 6.7% | 8.6% | 5.0% | 2.6% | 9.6% |  | 6.3% |  | 1.5% | 5.4% | 4.8% | 10.5% | 7.3% |
| **Stage** |  |  |  |  |  |  |  |  |  |  |  |  |  |  |  |
| localised to tissue or origin | 58.7% | 56.3% | 38.6% | 76.9% | 7.1% | 41.9% | 89.9% | 10.3% | 14.8% | 52.4% | 68.6% | 71.9% | 64.9% | 40.7% | 58.3% |
| regional spread, adjacent organs and/or regional lymph nodes | 12.0% | 36.8% | 45.4% | 13.4% | 0.7% | 32.3% | 4.0% |  | 2.4% | 23.8% | 9.0% | 14.9% | 18.4% | 23.9% | 21.9% |
| distant metastases | 4.0% | 2.6% | 7.0% | 4.3% | 0.7% | 14.2% | 1.1% |  | 0.3% | 14.3% | 0.9% | 2.3% | 5.7% | 9.8% | 3.6% |
| unknown | 25.3% | 4.2% | 8.9% | 5.4% | 91.5% | 11.6% | 5.0% | 89.7% | 82.5% | 9.5% | 21.5% | 10.9% | 11.0% | 25.6% | 16.1% |
| **Recent treatment** |  |  |  |  |  |  |  |  |  |  |  |  |  |  |  |
| No | 78.7% | 68.5% | 78.6% | 83.9% | 60.3% | 61.3% | 94.8% | 30.9% | 72.5% | 61.9% | 77.3% | 86.4% | 85.5% | 79.0% | 77.5% |
| Yes | 21.3% | 31.5% | 21.4% | 16.1% | 39.7% | 38.7% | 5.2% | 69.1% | 27.5% | 38.1% | 22.7% | 13.6% | 14.5% | 21.0% | 22.5% |

Diagnosis codes grouped under “other cancers” and the corresponding numbers of participants are included in Supplementary Table 1.
The numbers of participants with sex-specific cancers included 8 males with breast cancer. Those were excluded from subsequent prevalence ratio analyses.

**Table S4. Distribution of recent treatment by stage of cancer**

|  | **Recent treatment** | |  |
| --- | --- | --- | --- |
| **Stage of cancer** | No | Yes | Total |
| localised to tissue or origin | 4,087 | 773 | 4,860 |
|  | (84.1%) | (15.9%) | (100%) |
| regional spread | 218 | 610 | 1828 |
|  | (66.6%) | (33.4%) | (100%) |
| distant metastases | 141 | 160 | 301 |
|  | (46.8%) | (53.2%) | (100%) |
| unknown | 1015 | 329 | 1344 |
|  | (75.5%) | (24.5%) | (100%) |
| Total | 6,461 | 1,872 | 8,333 |

**Table S5. Comparison of crude versus adjusted (age and sex) prevalence for in paid workforce and not in paid workforce**

|  | **Crude prevalence** | **Adjusted (age and sex) prevalence** | **Adjusted (age and sex) PR** |
| --- | --- | --- | --- |
| **In paid workforce** |  |  |  |
| Any cancer | 58.1% | 61.8% | 0.91 |
| No cancer | 70.6% | 68.0% |  |
|  |  |  |  |
| **Not in paid workforce** |  |  |  |
| Any cancer | 41.9% | 30.4% | 1.18 |
| No cancer | 29.4% | 25.7% |  |

**Figure S1. Distribution of study population in types of workforce**

**
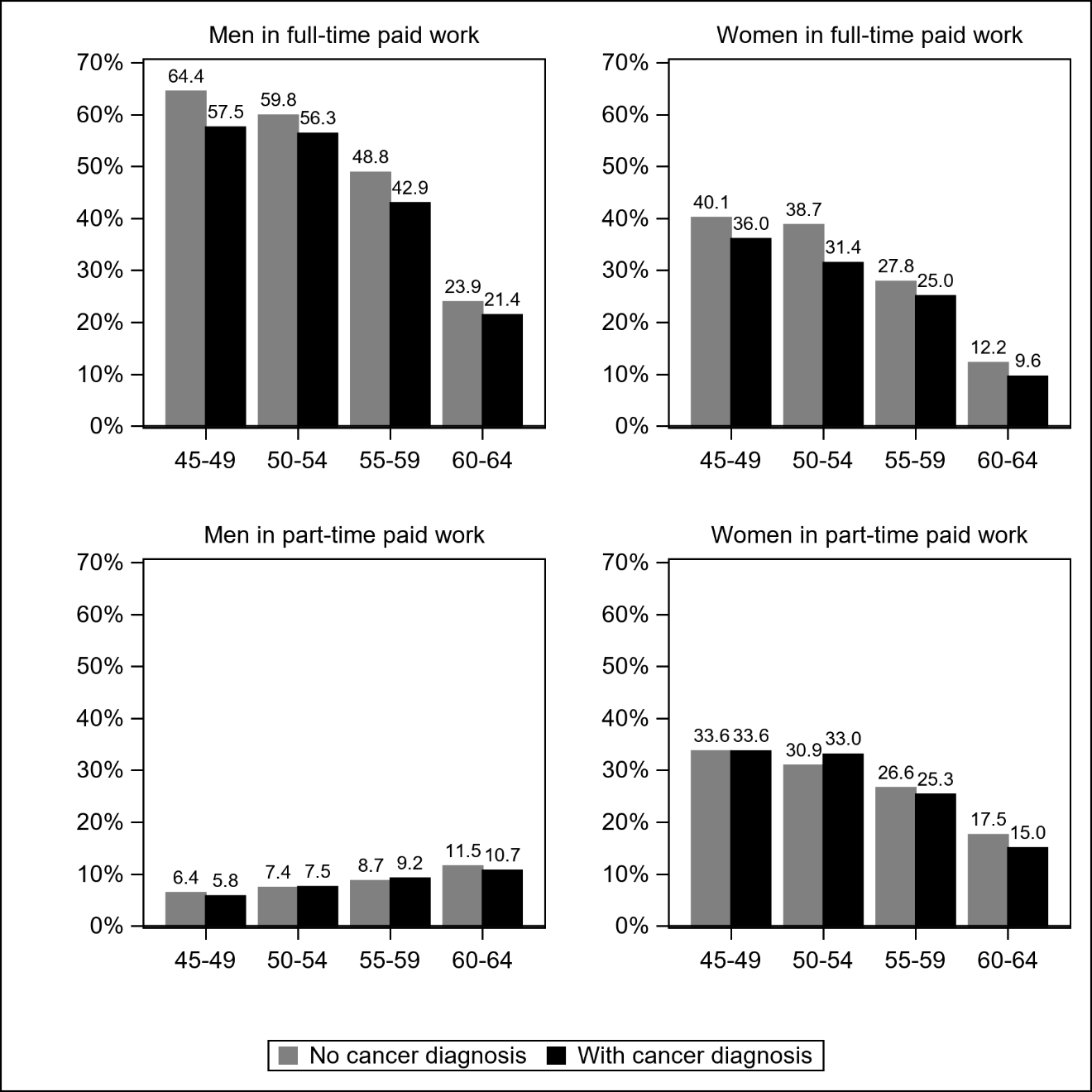
**

**Figure S2. Prevalence of and adjusted (age) prevalence ratios for being out of the paid workforce by cancer type and sex**


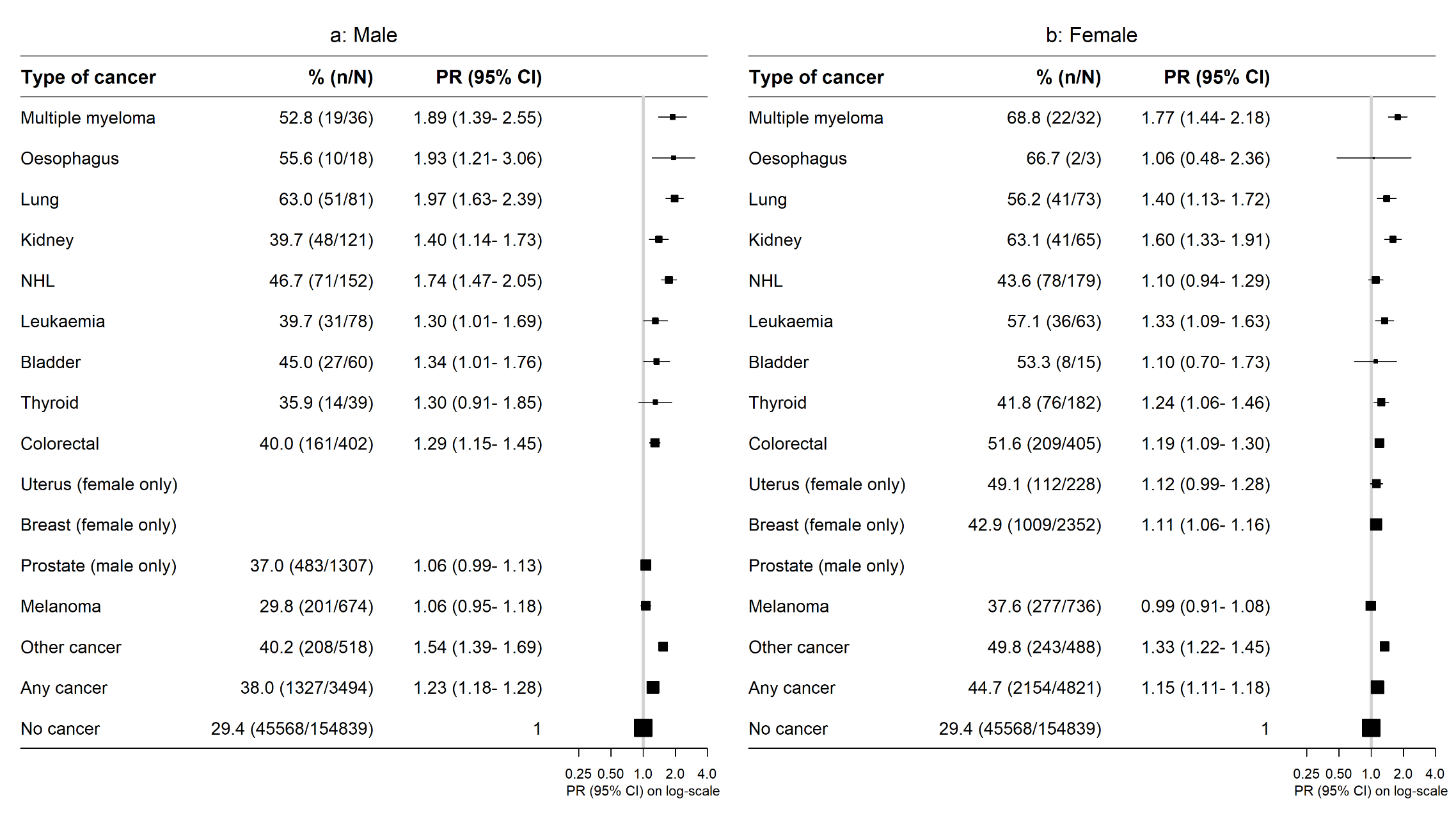


**Figure S3. Prevalence of and adjusted (sex) prevalence ratios for being out of the paid workforce by cancer type and age**

**
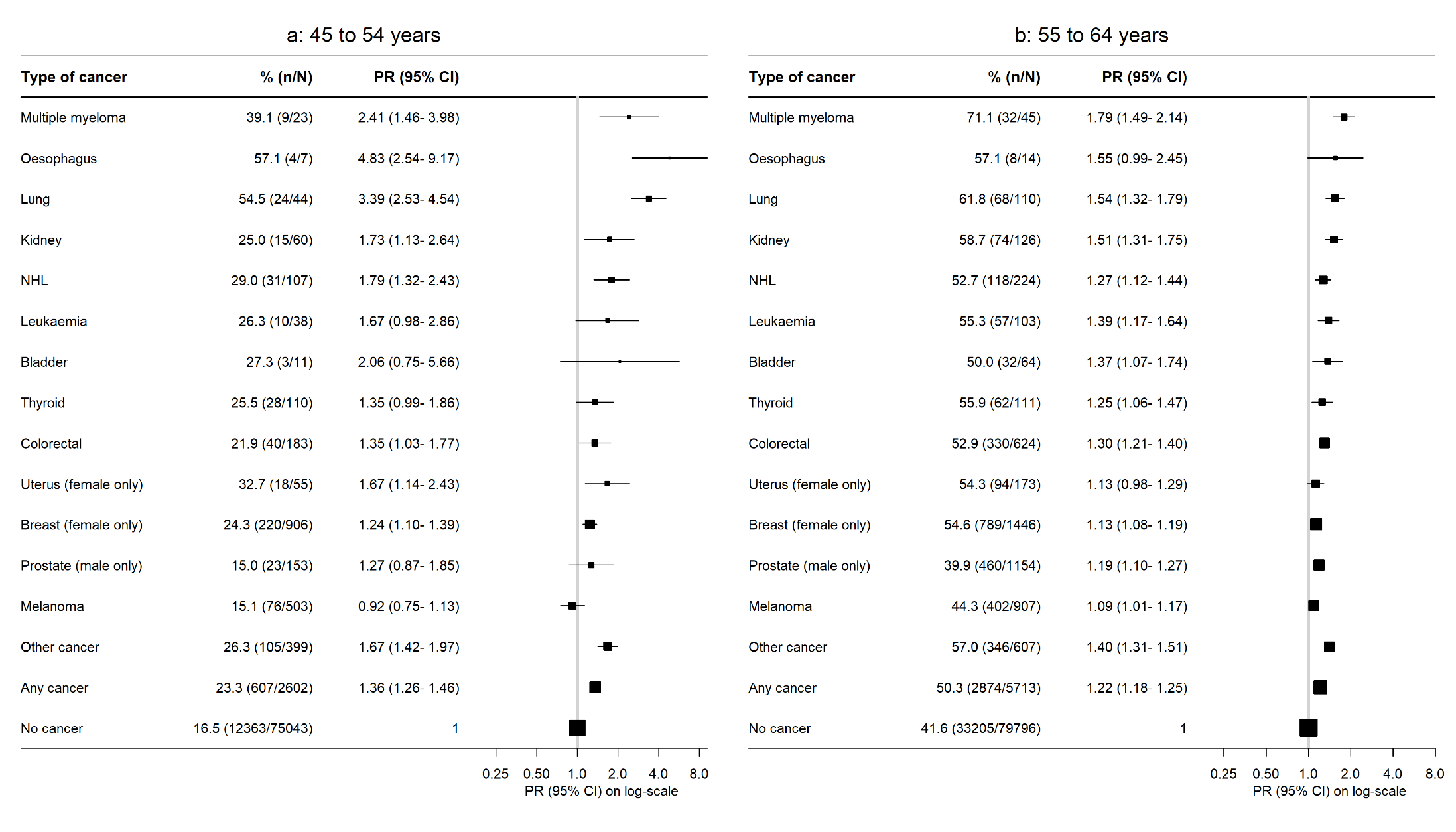
**

**Figure S4. Distribution of paid hours (0≤paid hours<100) per week among all participants**


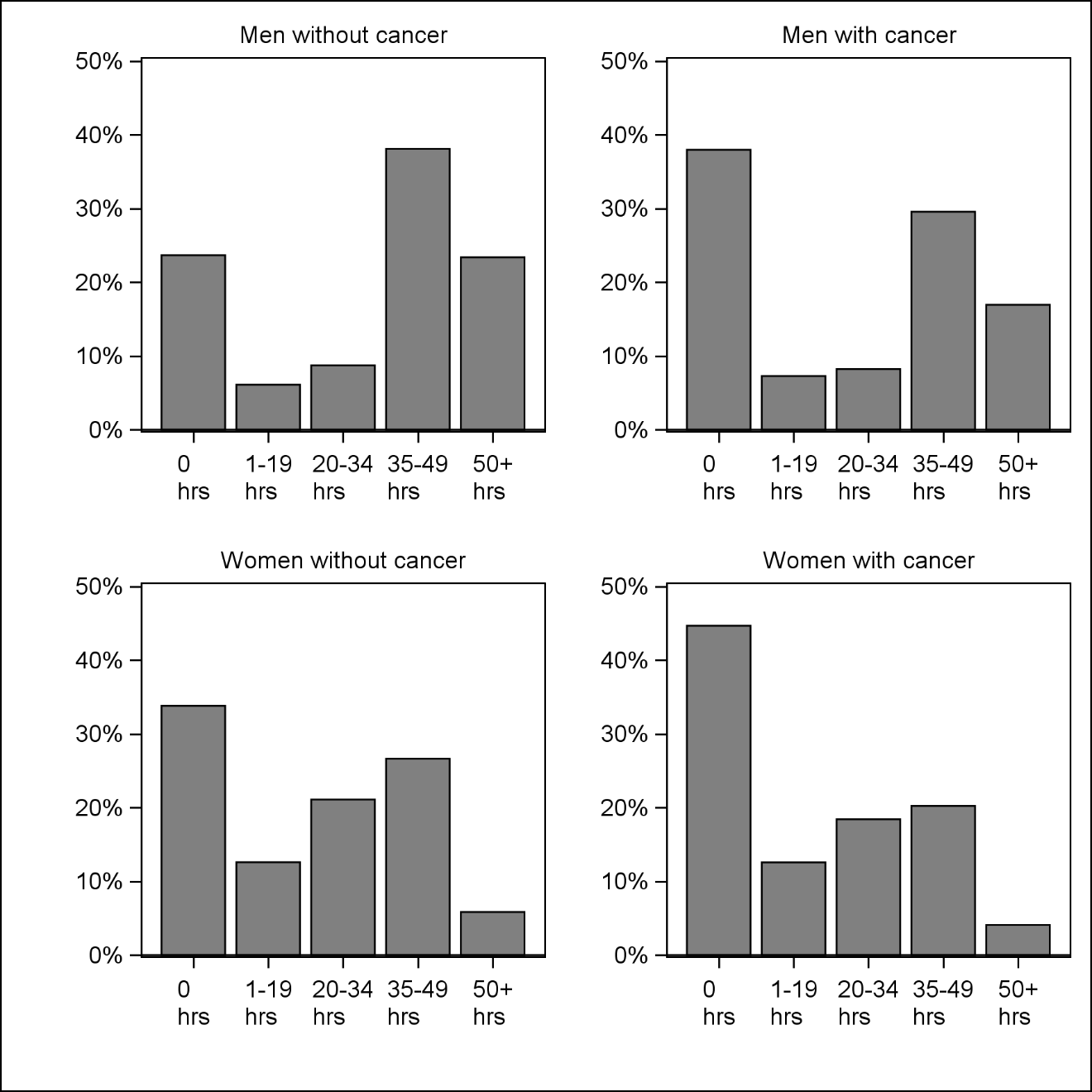


**Figure S5. Distribution of study population who are out of the paid workforce**

**
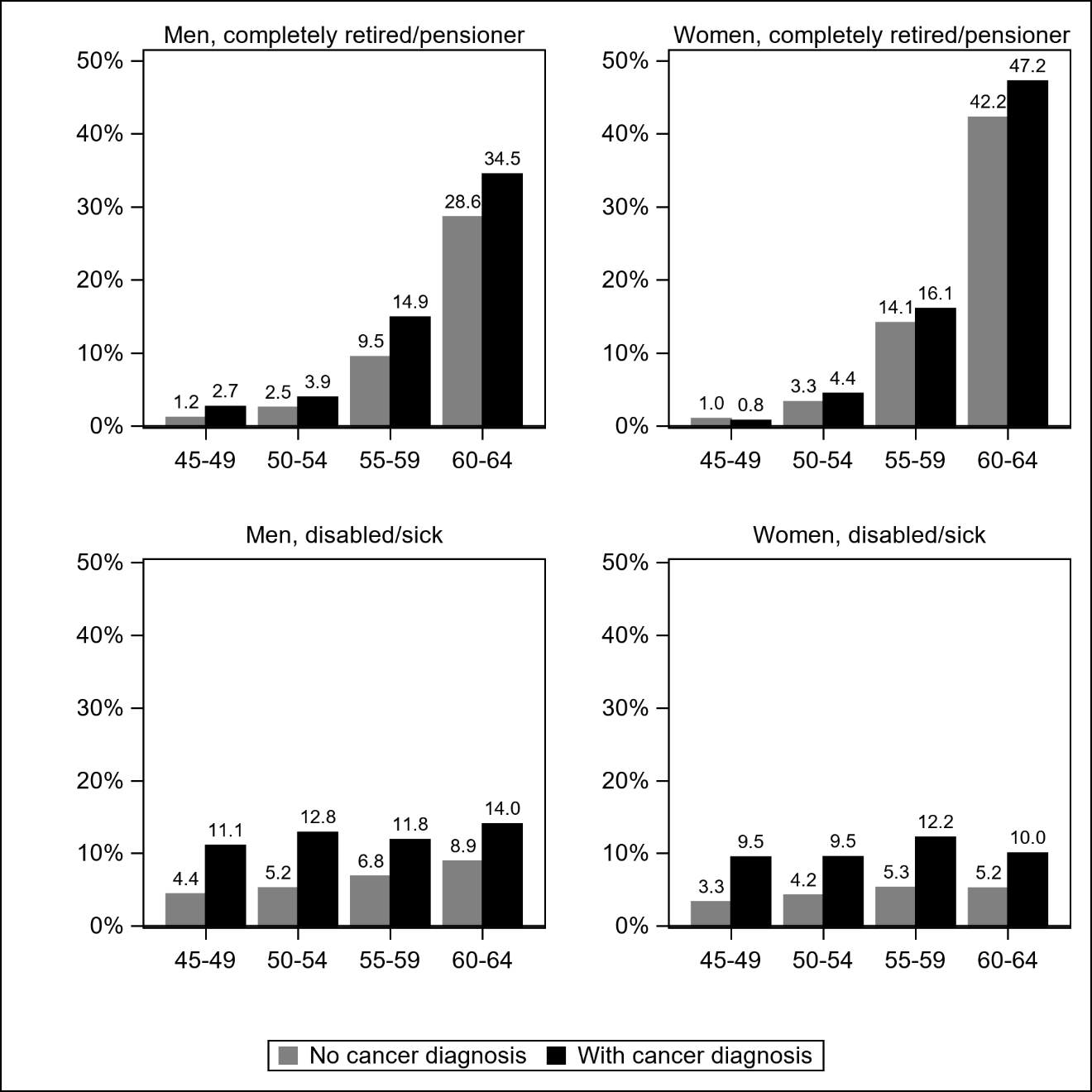
**

**Figure S6. Reasons for retirement among retired participants**


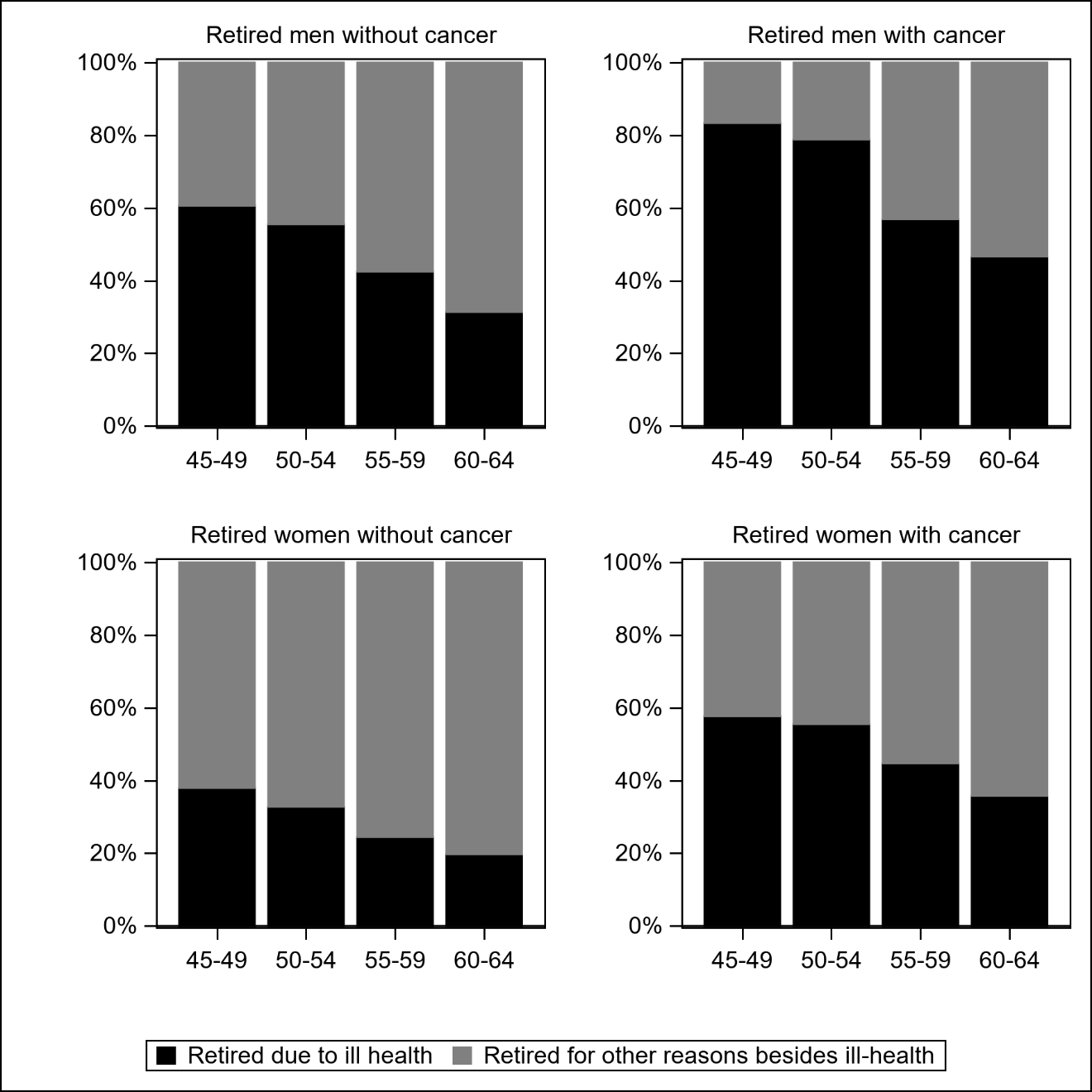


**Figure S7. Non-participation in the paid workforce (adjusted for age and sex)** **in various population subgroups**


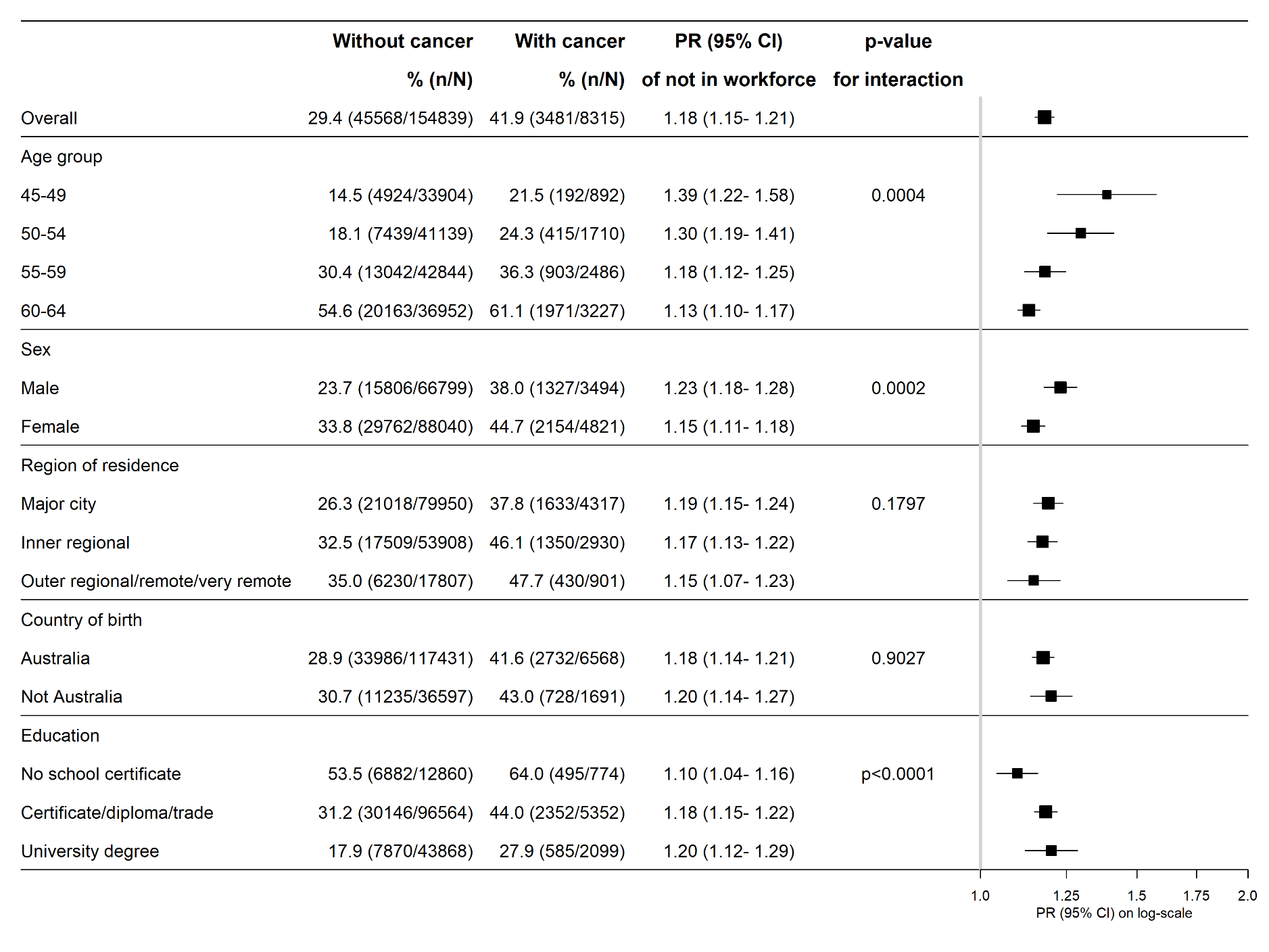


**Figure S8. Paid hours (0≤paid hours <100) of work per week (adjusted for age and sex) by cancer type, among all participants**


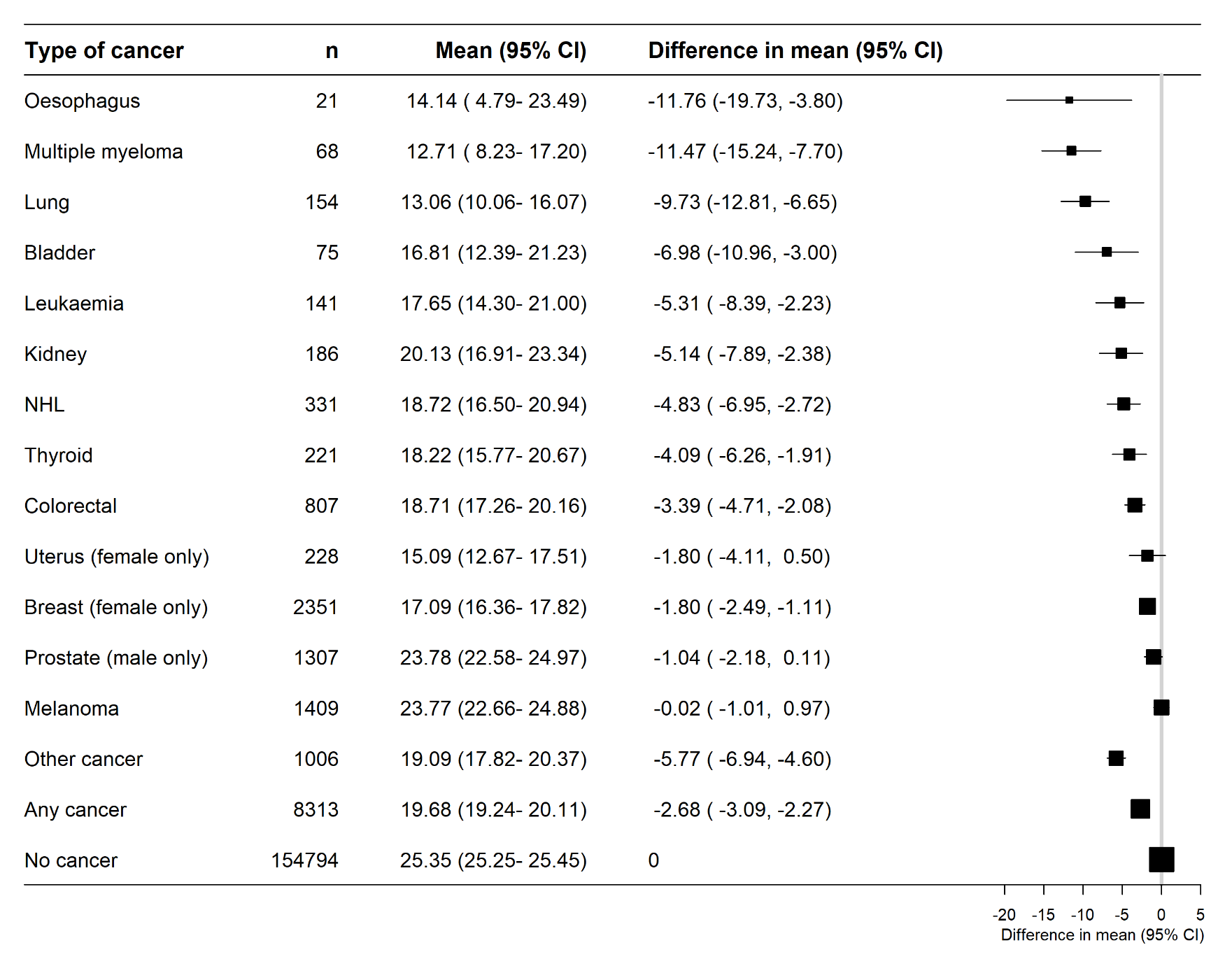


**Figure S9. Prevalence of and adjusted prevalence ratios for being out of the paid workforce by cancer type: sensitivity analysis with age as a continuous variable**

**
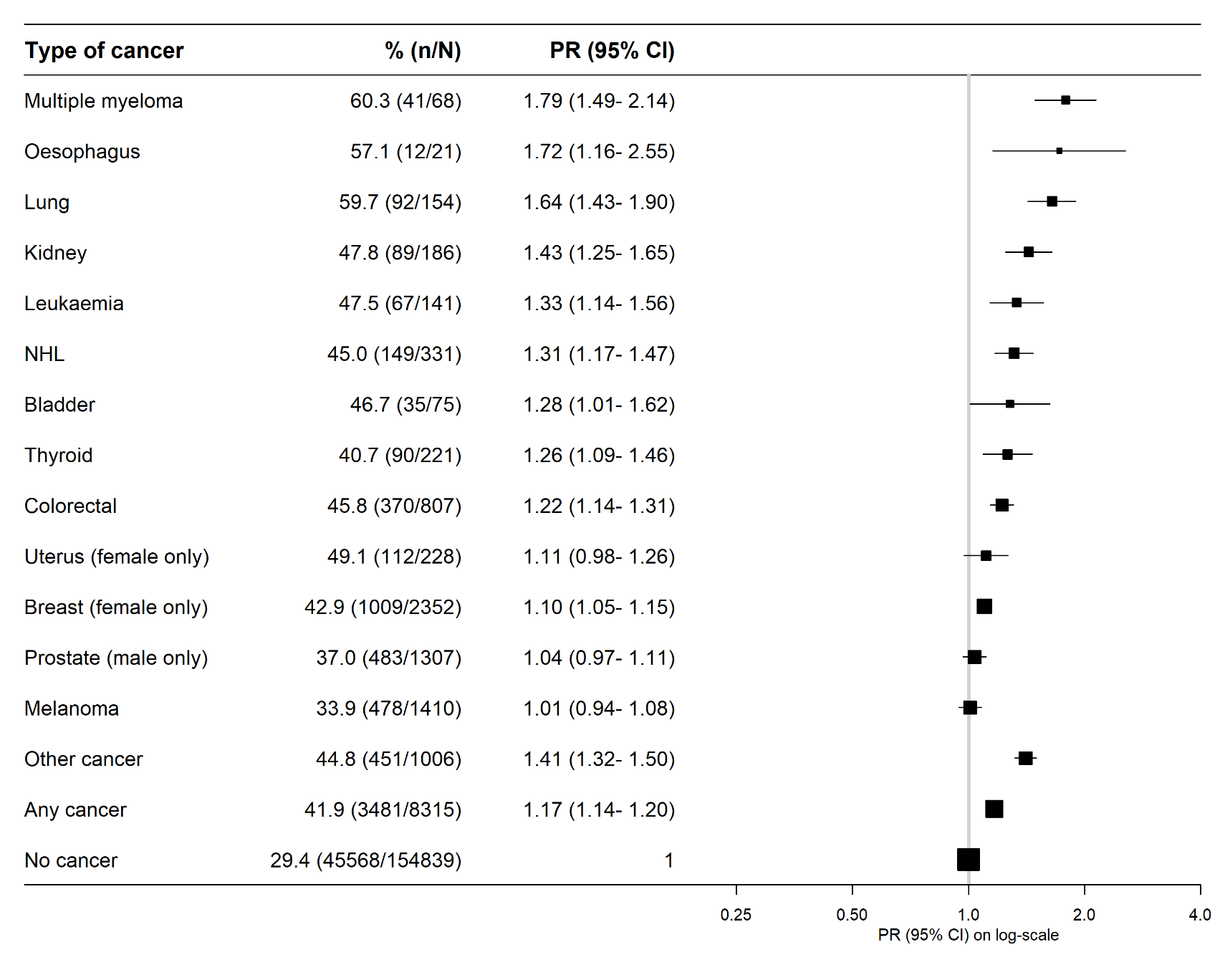
**

**Figure S10: Prevalence of and adjusted prevalence ratios for being out of the paid workforce according to joint categories of physical functioning limitations and cancer: sensitivity analysis with additional adjustment for education**

**
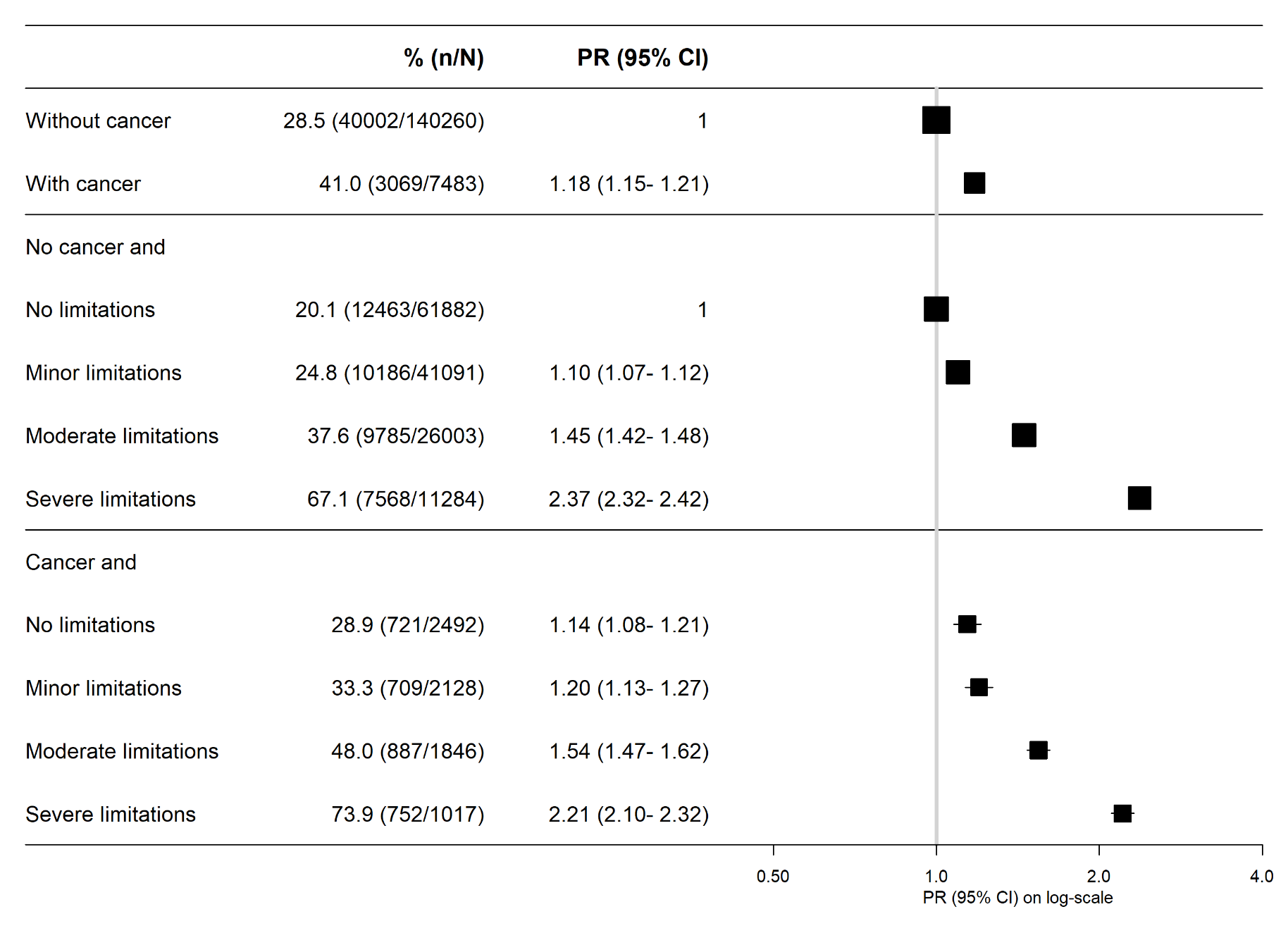
**

**Figure S11: Prevalence of and adjusted prevalence ratios for being out of the paid workforce according to joint categories of physical functioning limitations and cancer: sensitivity analysis with additional adjustment for region of residence**

**
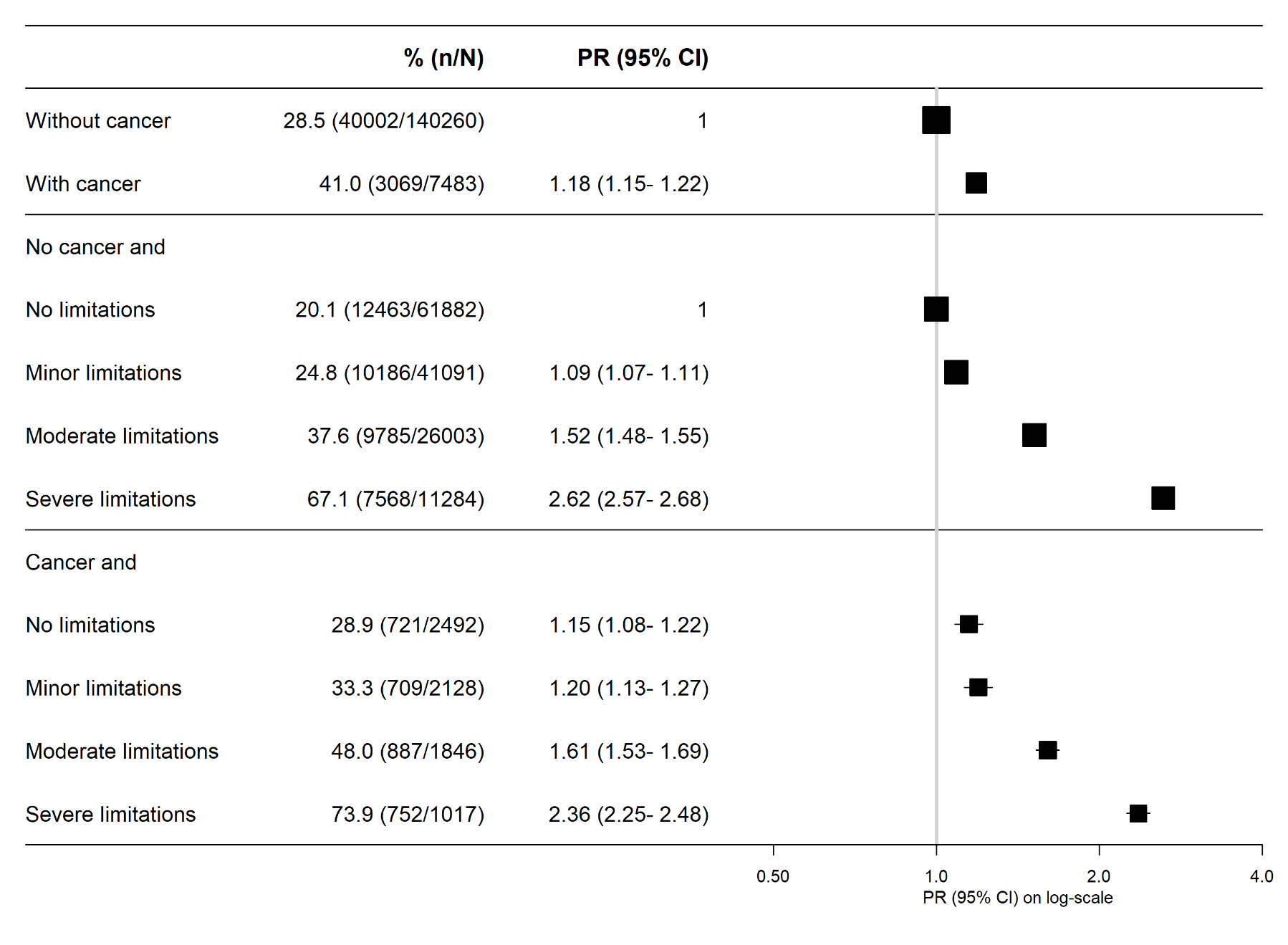
**
